# CAG-targeting artificial miRNA with reduced off-target risk for efficient lowering of pathogenic polyglutamine proteins

**DOI:** 10.1093/narmme/ugag023

**Published:** 2026-04-30

**Authors:** Marianna Pewinska-Kolodziejczak, Anna Kotowska-Zimmer, Lukasz Przybyl, Dorota Wronka, Anna Karlik, Michal Smuszkiewicz, Julia Balcerek, Joanna Suszynska-Zajczyk, Anna Piaszyk-Borychowska, Martyna Urbanek-Trzeciak, Gaurav Sablok, Emilia Kozlowska, Jan Podkowinski, Agnieszka Fiszer, Piotr Kozlowski, Luiza Handschuh, Marta Olejniczak

**Affiliations:** Department of Genome Engineering, Institute of Bioorganic Chemistry, Polish Academy of Sciences, Noskowskiego 12/14, 61-704 Poznan, Poland; Department of Genome Engineering, Institute of Bioorganic Chemistry, Polish Academy of Sciences, Noskowskiego 12/14, 61-704 Poznan, Poland; Laboratory of Mammalian Model Organisms, Institute of Bioorganic Chemistry, Polish Academy of Sciences, Noskowskiego 12/14, 61-704 Poznan, Poland; Laboratory of Mammalian Model Organisms, Institute of Bioorganic Chemistry, Polish Academy of Sciences, Noskowskiego 12/14, 61-704 Poznan, Poland; Laboratory of Mammalian Model Organisms, Institute of Bioorganic Chemistry, Polish Academy of Sciences, Noskowskiego 12/14, 61-704 Poznan, Poland; Department of Genome Engineering, Institute of Bioorganic Chemistry, Polish Academy of Sciences, Noskowskiego 12/14, 61-704 Poznan, Poland; Department of Genome Engineering, Institute of Bioorganic Chemistry, Polish Academy of Sciences, Noskowskiego 12/14, 61-704 Poznan, Poland; Department of Biochemistry and Biotechnology, Poznan University of Life Sciences, 60-632 Poznan, Poland; Department of Genome Engineering, Institute of Bioorganic Chemistry, Polish Academy of Sciences, Noskowskiego 12/14, 61-704 Poznan, Poland; Department of Molecular Genetics, Institute of Bioorganic Chemistry, Polish Academy of Sciences, Noskowskiego 12/14, 61-704 Poznan, Poland; Laboratory of Genomics, Institute of Bioorganic Chemistry, Polish Academy of Sciences, Noskowskiego 12/14, 61-704 Poznan, Poland; Department of Medical Biotechnology, Institute of Bioorganic Chemistry, Polish Academy of Sciences, Noskowskiego 12/14, 61-704 Poznan, Poland; Laboratory of Genomics, Institute of Bioorganic Chemistry, Polish Academy of Sciences, Noskowskiego 12/14, 61-704 Poznan, Poland; Department of Medical Biotechnology, Institute of Bioorganic Chemistry, Polish Academy of Sciences, Noskowskiego 12/14, 61-704 Poznan, Poland; Department of Molecular Genetics, Institute of Bioorganic Chemistry, Polish Academy of Sciences, Noskowskiego 12/14, 61-704 Poznan, Poland; Laboratory of Genomics, Institute of Bioorganic Chemistry, Polish Academy of Sciences, Noskowskiego 12/14, 61-704 Poznan, Poland; Department of Genome Engineering, Institute of Bioorganic Chemistry, Polish Academy of Sciences, Noskowskiego 12/14, 61-704 Poznan, Poland

## Abstract

Huntington’s disease (HD) is the best-known example of a neurodegenerative disorder caused by the expansion of a glutamine-encoding CAG repeat in the causative gene. Growing evidence indicates that somatic CAG expansions play a key role in disease progression, providing a strong rationale for therapeutic strategies directly targeting the repeat tract. However, achieving sufficient efficacy while maintaining allele selectivity and minimizing off-target effects remains a major challenge. Here, we developed allele-selective, CAG-targeting artificial microRNA (amiRNA) molecules that exhibit significantly reduced off-target risk. This was achieved by introducing specific substitutions at selected positions within the guide strand. These molecules effectively downregulated polyglutamine (polyQ) proteins in cellular models of HD, spinocerebellar ataxias types 1 and 3, and dentatorubral pallidoluysian atrophy. The most promising candidate, amiR136-13A, reduced mutant huntingtin levels in different brain regions of the HD mouse model and did not induce toxicity up to 28 weeks following a single administration of an AAV5 vector. Transcriptomic profiling of human HD neural stem cells treated with amiR136-13A revealed minor changes in gene expression. Moreover, amiR136-13A reduced the level of HTT1a, a short pathogenic isoform of huntingtin. Collectively, these findings identify amiR136-13A as a potent, selective, and safe therapeutic candidate for HD and potentially other polyQ disorders.

## Graphical Abstract

**Graphical Abstract:**
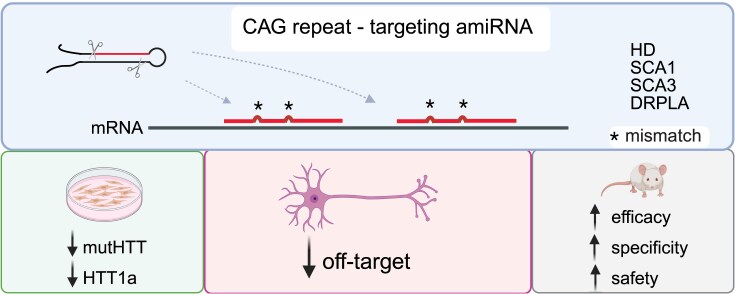

## Introduction

The expansion of microsatellite CAG repeat in the coding regions of unrelated genes is a hallmark of a group of inherited neurological disorders known as polyglutamine (polyQ) diseases, which include Huntington’s disease (HD), dentatorubral-pallidoluysian atrophy (DRPLA), spinobulbar muscular atrophy (SBMA), and spinocerebellar ataxias (SCAs) [1, 2]. The elongated CAG tract is translated into an abnormally long polyQ domain, which causes protein misfolding and aggregation. PolyQ proteins perform important cellular functions that, due to mutations, are disrupted through both toxic gain-of-function and loss-of-function mechanisms [3–5]. This leads to progressive neuronal degeneration and atrophy of disease-specific brain areas. In the case of HD, an expanded CAG tract (>36 repeats) is located in the first exon of the huntingtin gene (*HTT*) [6–8]. Its length inversely correlates with the age at onset and severity of symptoms, which include motor and cognitive dysfunction, as well as psychiatric disturbances [9]. Additionally, longer repeats are susceptible to somatic instability over time, thereby increasing their inherited length [10]. Massive expansion of CAG repeats has been observed in selected tissues (brain and liver) and cell types (medium spiny neurons) in HD patients [11–13]. Repeat expansion also affects the abnormal processing of *HTT* pre-mRNA, resulting in the formation of the highly pathogenic HTT1a protein (encoded by *HTT* exon 1) [14, 15]. The presence of HTT1a has been well documented in post-mortem HD brains and in various transgenic HD mouse models [16, 17]. Accumulation of this toxic fragment over time is believed to contribute significantly to cellular dysfunction and disease progression in HD [18, 19].

To date, no disease-modifying treatment has been approved for HD, as well as other polyQ disorders. Among many therapeutic strategies, HTT-lowering approaches using antisense oligonucleotides (ASOs), RNA interference (RNAi) triggers, zinc finger transcriptional repressors, or small molecules are at the most advanced stage [20–22]. Reduction of the HTT protein level alleviates motor and neuropathological abnormalities in animal models of HD. However, they require continuous refinement, as three promising clinical trials using ASOs have been terminated due to side effects (NCT03761849) or low efficacy (NCT03225833 and NCT03225846). One challenge of this strategy is to achieve selectivity in lowering mutant protein levels, as increasing evidence suggests a role for normal HTT not only in early embryogenesis and the development of the central nervous system but also in adult life [23–25]. Preferential silencing of mutant *HTT* expression has been achieved in cellular and mouse models of HD using RNAi triggers that directly target the CAG repeats in transcripts [26–29]. It takes advantage of the ability of more silencing complexes to bind longer CAG sequences and act in a cooperative manner [30, 31]. The introduction of sequence modifications into CAG-targeting siRNAs, which create mismatches with the messenger RNA (mRNA) target, shifts the mechanism of action from transcript cleavage to translation inhibition and/or deadenylation (miRNA-like mechanism). Ribosomes readily remove the mismatched RNA-induced silencing complex (RISC) from normal transcripts but are less efficient at displacing complexes formed by multiple RISCs on mutant transcripts [32]. Moreover, CAG-targeting siRNA in the form of short hairpin RNA (shRNA) or artificial microRNA (amiRNA) can be efficiently delivered to the brain *via* a viral vector, ensuring long-term expression after a single administration [33–36]. Previously, we developed vectorized CAG-targeting RNAi tools and demonstrated that, unlike shRNA, amiRNA did not induce toxicity in a mouse model of HD [26]. A single intrastriatal injection of AAV5 carrying amiR136-A2 resulted in a significant and selective reduction in the levels of mutant HTT and polyQ aggregates in the striatum. Despite these encouraging results, several issues still require improvement, including the need for high doses to achieve efficient *HTT* silencing *in vivo*, the risk of off-target effects, and the low level of *HTT* silencing in the cerebral cortex.

Therefore, we aimed to develop amiRNA with improved characteristics, combining high efficacy with specificity. Following a detailed analysis of potential off-target sites in the human genome, we proposed sequence modifications to the amiR136-A2 molecule that significantly reduce the risk of nonspecific binding. Newly designed amiRNAs effectively downregulated polyQ proteins in cellular models of HD, SCA1, SCA3, and DRPLA. The most promising candidate, amiR136-13A, preferentially reduced mutHTT levels in different brain regions of the HD mouse model, including the cerebral cortex. This allowed the therapeutic dose to be reduced by half compared to amiR136-A2 without compromising efficacy. Importantly, no toxicity was observed up to 28 weeks after a single injection of an AAV5 vector. Transcriptomic analysis of human HD neural stem cells (NSCs) treated with amiR136-13A revealed small changes in gene expression, which partially showed the desired shift toward expression observed in healthy cells. Finally, as a proof of concept, we demonstrated that amiR136-13A reduces the level of HTT1a and the number of its cellular aggregates, which further strengthens its therapeutic potential. These results suggest that improved CAG-targeting amiRNA may be promising candidates for allele-selective therapy of HD and other polyQ diseases.

## Materials and methods

### Bioinformatic mapping and BLAST analysis

The sequence of the siRNA A2 guide strand (5′ CUGCUGCAGCUGCUGCUGC 3′) was mapped to the human genome using Bowtie (v.1.2.3). The hg38 dataset from the UCSC Genome Browser (University of California, Santa Cruz), released on 10 August 2018, and available at https://hgdownload.soe.ucsc.edu/goldenPath/hg38/bigZips/latest/hg38.fa.gz. The NCBI Nucleotide BLAST algorithm was used to estimate the number of potential off-target binding sites in the human transcriptome.

### Cell culture

Fibroblasts from HD patient (GM04281; 68/17Q), SCA1 patient (GM06927; 52/29Q), SCA3 patient (GM06153; 69/18Q), and DRPLA patient (GM13717; 65/15Q) were obtained from Coriell Cell Repositories (Camden, NJ) and grown in minimal essential medium (MEM) (Sigma–Aldrich, St. Louis, MO) supplemented with 10% fetal bovine serum (FBS) (Sigma–Aldrich), 2 mM *L*-glutamine (Sigma–Aldrich), and 1× antibiotics (Sigma–Aldrich). HEK293T cells were grown in Dulbecco’s modified Eagle’s medium (DMEM) (Sigma–Aldrich) supplemented with 8% FBS, penicillin–streptomycin (Pen-Strep), and 2 mM *L*-glutamine. Flp-In T-Rex -293 (Invitrogen) were grown in DMEM supplemented with 10% FBS, 0.5× Pen-Strep (Gibco), 2 mM *L*-glutamine, and 5 ug/ml blasticidin S (Gibco). Human HD induced pluripotent stem cells (iPSCs) (ND42222, NINDS Repository) and previously generated isogenic control IC39 [21] were grown in StemFlex medium (Gibco) with 0.5× Pen-Strep.

### iPSCs differentiation into NSCs

iPSCs were differentiated into NSCs using the STEMdiff SMADi Neural Induction Kit (STEMCELL Technologies) and a monolayer protocol, following the manufacturer’s guidelines and as previously described [3]. After the fourth passage, NSC identity was confirmed by the expression of the markers *SOX1* (SRY-box transcription factor 1), *SOX2* (SRY-box transcription factor 2), and *PAX6* (paired box 6), as well as by a reduction in the expression of the pluripotency marker *OCT4* (octamer-binding transcription factor 4), as determined by reverse transcription quantitative polymerase chain reaction (RT-qPCR).

### Plasmids and viral vectors

The shRNA and amiRNA expression cassettes were generated from DNA oligonucleotides (Metabion and Integrated DNA Technologies, respectively; see the sequences in Supplementary Table S1). Pairs of oligonucleotides were annealed and ligated into the pGreenPuro (for shRNAs) or pCDH-CMV-MCS-EF1-Puro (for amiRNAs) (System Biosciences, Palo Alto, CA) expression plasmid and verified through sequencing. For lentivirus production, the plasmids were cotransfected with the packaging plasmids pCMV-VSV-G and pCMV-dR8.2 dvpr (Addgene) into HEK293T cells using polyethyleneimine, according to the manufacturer’s protocol. The medium was collected 48 and 72 h after transfection, and the viral supernatants were passed through 0.45-µm filters and concentrated using PEG-it Virus Precipitation Solution (System Biosciences). The lentiviral vectors were resuspended in Opti-MEM (Gibco), and the virus titers (TU/ml) were determined through flow cytometry (Attune NxT, Invitrogen) based on copGFP expression. Transduction of fibroblasts and NSCs was performed at an MOI of 10 in the presence of polybrene (4 mg/ml). Total protein was harvested 7 days post-transduction. The Scramble (SCR, in terms of shRNAs) or luciferase-targeting (LUC, in terms of amiRNAs) construct was used as a negative control. For *in vivo* experiments, AAV5 vectors were produced by Virovek (Texas, USA). The amiRNA was expressed under the control of the CAG Pol II promoter, and an empty vector was used as a control. The pcDNA™5/FRT/TO plasmid (Thermo Fisher Scientific), dedicated to the Flp-In™ T-REx™ system (Thermo Fisher Scientific), was used to express the truncated HTT1a protein under the control of the CMV promoter.

### Flp-In T-REx -293 system

An expression cassette containing *HTT* exon 1 with 83 CAG repeats, a Kozak sequence, STOP codons, and a triple FLAG tag was cloned from a custom-designed pUC57 (GenScript) into the pcDNA™5/FRT/TO plasmid using *PmeI* and verified by sequencing. Flp-In T-REx -293 cells were cotransfected with pcDNA5/FRT/TO-HTT1a and pOG44 using Lipofectamine 2000. Stable integrants were selected using hygromycin B (100 μg/ml) with the addition of blasticidin (5 μg/ml). Expression of *HTT1a* was induced with doxycycline (1 μg/ml), and the following day, cells were transduced with viral particles at an MOI of 10 in medium containing 2% FBS, antibiotics, and polybrene (4 μg/ml). After 18 h, the medium was replaced, and on day 4 post-transduction, cells were harvested for RNA and protein isolation or immunohistochemical analysis.

### MTT assay

HEK293T cells were seeded into 96-well plates. After 24 h, the cells were transfected with plasmids encoding amiRNA using Lipofectamine. Plasmid DNA was applied at doses of 10, 50, or 100 ng per well. After 4 h, the transfection medium was replaced with complete growth medium. Forty-eight hours after transfection, the medium was removed, and 100 µl of MTT-containing medium at a final concentration of 0.5 mg/ml was added to each well. Cells were incubated with MTT for 2 h at 37°C. After incubation, the MTT solution was removed, and the resulting formazan crystals were resuspended in dimethyl sulfoxide (DMSO). Absorbance was measured using a VICTOR Nivo multimode plate reader.

### Luciferase assays

Reporter assays were done as previously [26]. Briefly, HEK293T cells were cultured in 24-well plates and cotransfected with HTT reporter plasmids (containing 16, 57, or 85 CAG repeats; encoding Renilla and firefly luciferase) and shRNA/amiRNA constructs (5–500 ng) using Lipofectamine 2000. After 48 h, cells were lysed using Passive Lysis Buffer (Promega), and luciferase activity was measured with the Dual-Luciferase Reporter Assay (Promega) on a Victor 4 reader (PerkinElmer). A plasmid with a scrambled sequence or an empty plasmid served as the control (for shRNA and amiRNA, respectively); firefly luciferase signals were normalized to Renilla luciferase signals.

### RNA isolation and RT-qPCR

Total RNA from HEK293T and mouse striata was isolated using TRIzol Reagent (Thermo Fisher Scientific) and Phenol equilibrated, stabilized chloroform: isoamyl alcohol (25:24:1) (PanReac Applichem, Barcelona, Spain). Total RNA from NSCs was isolated 7 days post-transduction using the PicoPure RNA Isolation Kit (Thermo Fisher Scientific). Total RNA from HEK293 Flp-In T-Rex cells was isolated using the Direct-zol RNA Miniprep Kit (Zymo Research). A DeNovix Nanodrop Spectrophotometer was used to measure the RNA concentration. A total of 500 ng of total RNA was transcribed to cDNA using SuperScript III Reverse Transcriptase (Invitrogen) at 55ºC. RT-qPCR was performed in the CFX Connect Real-Time PCR Detection System (Bio-Rad, Hercules, CA) using SsoAdvanced Universal SYBR Green Supermix (Bio-Rad) with *β-actin* or *EEF2* (for NSCs and Flp-In T-Rex -293 cells) as the reference gene under the following thermal cycling conditions: denaturation at 95ºC for 30 s followed by 40 cycles of denaturation at 95ºC for 15 s and annealing at 60ºC for 30 s. Sequences of specific primers are listed in Supplementary Table S2.

### Protein isolation and western blotting

Proteins from cell cultures and mouse tissues were isolated using PB buffer (60 mM Tris–base, 2% sodium dodecyl sulfate (SDS), 10% sucrose, 2 mM phenylmethylsulfonyl fluoride (PMSF)) and incubated at 95°C for 5 min. For the separation of proteins isolated from DRPLA fibroblasts, NSCs, and mouse tissues, commercial NuPAGE™ Tris–Acetate Mini Protein Gels (Invitrogen) 3%–8% were used. Proteins isolated from HD fibroblasts were separated on Tris–acetate SDS–polyacrylamide gel (1.5 cm, 4% stacking gel/4.5 cm, 5% resolving gel, acrylamide:bis-acrylamide ratio of 49:1) in XT Tricine buffer (Bio-Rad). Proteins isolated from SCA3 fibroblasts were separated on Tris–acetate SDS–polyacrylamide gel (5% stacking/12% resolving gel) in NuPAGE Tris–Actetate SDS running buffer, while proteins from SCA1 fibroblasts were separated on Bis–Tris 4%–12% gel (Thermo Fisher Scientific) in Nu-Page MOPS SDS running buffer. A total of 30 μg of protein mixed with loading dye was loaded onto the gel. Proteins were transferred to a nitrocellulose membrane (Cytiva) by wet transfer using the Mini Trans-Blot Cell system (Bio-Rad) either overnight (18 h at 30 V) or for 1 h at 100 V. Primary and secondary antibodies, along with their dilutions, are listed in Supplementary Table S3. Immunoreactions were detected using the Westar Antares substrate (Cyanogen). Protein bands were scanned directly from the membrane using a camera, and band densities were quantified with the Gel-Pro Analyzer software (Media Cybernetics). Plectin, GAPDH, vinculin, or calnexin was used as a loading control.

### Immunofluorescence

Flp-In T-Rex -293 cells were fixed with 4% paraformaldehyde (directly on coverslips) for 10 min and washed three times with PBS. Next, the cells were permeabilized with 0.25% Triton X-100 for 10 min and blocked with 3% bovine serum albumin (BSA) in PBS-T for an additional 30 min. The monoclonal mouse anti-FLAG M2 primary antibody (Sigma–Aldrich) was diluted 1:200 in 1% BSA in PBS and incubated with the cells at room temperature for 2 h. After thorough washing, cells were incubated with Alexa Fluor 594 donkey anti-mouse (Jackson ImmunoResearch) secondary antibody diluted 1:1000 in 1% BSA in PBS at room temperature for 45 min. Finally, the coverslips were mounted on glass slides using SlowFade Diamond Antifade Mountant with DAPI (Thermo Fisher Scientific), and fluorescence images were obtained using a Keyence BZ-X800 microscope with a 60× oil-immersion objective. This experiment was performed in four independent biological replicates. At least five randomly selected points on each coverslip were imaged and analyzed using ImageJ/Fiji (Wayne Rasband and contributors, National Institutes of Health, USA). Cell nuclei were identified based on DAPI staining-positive signals. Images underwent contrast enhancement followed by automatic thresholding using the Otsu method. Binary masks were generated, and nuclei were separated using the Watershed algorithm. Regions of interest corresponding to nuclei were defined, and particle analysis was performed with the Analyze Particles command. In the subsequent step, Alexa Fluor 594-positive signals corresponding to HTT aggregates were manually quantified using the Multi-point tool in ImageJ. This analysis enabled the determination of the number of cell nuclei and HTT aggregates in cells transduced with control (amiR136-LUC) compared to amiR136-13A.

### HD animal model

As previously, we employed the YAC128 transgenic mouse model of HD [FVB-Tg(YAC128)53Hay/J] (Jackson Laboratory). This model carries the full-length human *HTT* gene, with exon 1 containing 125 CAG repeats interrupted by 9 CAA triplets. As a reference group in some analyses, healthy FVB/NJ mice (Jackson Laboratory) were used. Mice were bred in the animal facility of the Center for Advanced Technologies, Adam Mickiewicz University in Poznan, Poland (CAT AMU). Animals were housed in individually ventilated cages with access to water and food *ad libitum*. All of the experiments were approved by the Local Ethical Committee for Animal Experiments in accordance with resolution no. 45/2018 and 17AiB/2022.

### Surgical procedures

Single bilateral injections into the striatum were performed under inhalation anesthesia, using the following stereotactic coordinates relative to bregma: AP +0.7 mm, ML ±1.7 mm, DV –3.5 mm (3 µl per hemisphere). The amiRNA was delivered using an adeno-associated viral vector of serotype 5 (AAV5), which exhibits enhanced tropism for neuronal cells. The vectors were produced by Virovek. Control animals received a vector lacking amiRNA (referred to as an “empty” vector). Two doses were used—low and high (see Supplementary Table S4).

### Behavioral tests

Behavioral tests are described in the Supplementary Material.

### Tissue harvesting

Mice were sacrificed by cervical dislocation 11 weeks (short-term) or 28 weeks (long-term) post-injection. The striatum, hippocampus, and cortex were dissected, and each hemisphere was divided for the isolation of DNA, RNA, and protein. In the long-term study, blood was collected for serum preparation. All samples were immediately frozen at −80°C.

### Determination of NfL levels

Serum NfL concentration was measured 28 weeks post-injection. The analysis included mice treated with low or high doses, empty vector, as well as untreated healthy and diseased controls. Measurements were performed by RayBiotech (USA) using the Simoa Assay, with each sample analyzed in triplicate.

### Small RNA next-generation sequencing and data analysis

Small RNA next-generation sequencing, preceded by RNA library preparation, was performed using material derived from HEK293T cells and striatal tissue from YAC128 mice. RNA sequencing from HEK293T cells was conducted at the Center of New Technologies, University of Warsaw, while mouse RNA sequencing was performed by Novogene (Germany). In both cases, an Illumina NovaSeq 6000 platform was used. For the HEK293T samples, 2 μg of total RNA was sequenced to a depth of 10 million reads. For the mouse samples, 2 μg of total RNA was sequenced to a depth of 30 million reads. Bioinformatic analysis was performed by Data2biology.

### Next-generation mRNA sequencing and data analysis

Next-generation mRNA sequencing from NSCs was performed using the Illumina NovaSeq 6000 platform at the IBCH PAS. The NGS library was generated using the KAPA RNA HyperPrep Kit with RiboErase (HMR) and the KAPA Unique Dual-Indexed Adapter Kit. For each sample, 0.5 μg of total RNA was used, and sequencing was carried out to a depth of 100 million reads. Demultiplexing was performed using bcl2fastq, with the index2 sequence reversed. After demultiplexing, read quality was assessed and cleaned using FASTQC (https://www.bioinformatics.babraham.ac.uk/projects/fastqc/) and FastP [23]. The reads were then aligned to the human genome (GRCh38) using the STAR aligner [37]. Aligned reads in BAM format were subsequently sorted and indexed using SAMtools [38]. For differential expression analysis, raw read counts were processed using DESeq2 [39]. Principal component analysis (PCA) was conducted using the PCAtools R package [40]. Bubble plots were generated using the SRplot tool (https://www.bioinformatics.com.cn/en). All computations were performed on the HPC server provided by Poznań Supercomputing and Networking Center (PCSS).

### Statistical analysis

Each experiment was performed in at least three independent biological and three technical replicates, except for the analysis of neuronal precursor markers, which was carried out in two biological replicates. Details of the statistical tests are provided in the figure legends. Statistical analyses were performed using Prism (GraphPad).

## Results

### Introduction of two mismatches to CAG-targeting RNAs significantly reduces the number of potential off-targets while maintaining their silencing activity

**Figure 1.**
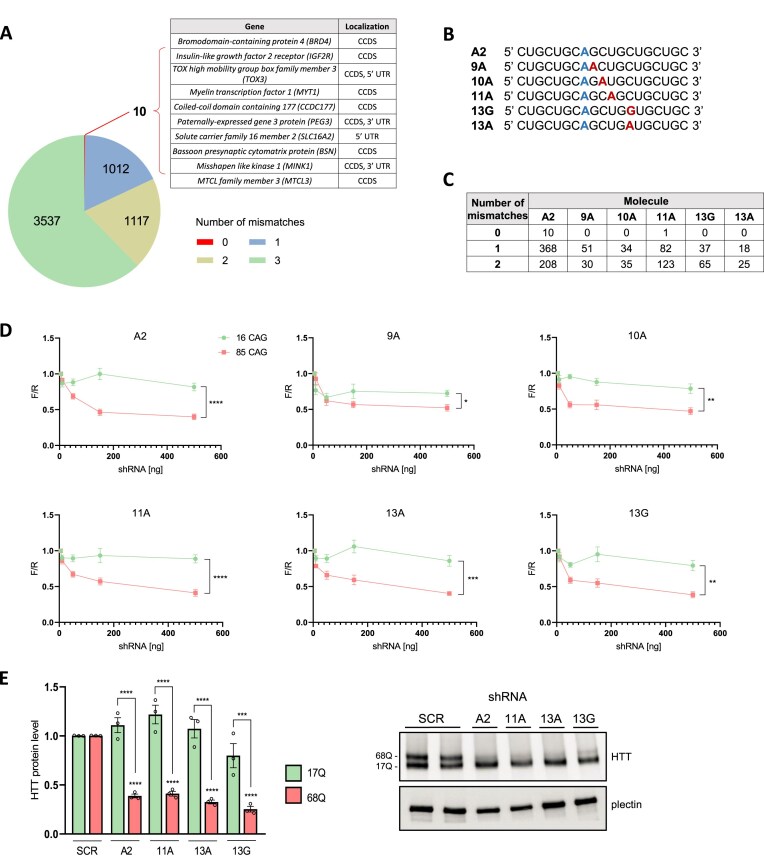
Design and efficiency analysis of new CAG-targeting shRNAs. (**A**) Circular diagram showing the number of potential off-target sites in the human genome with 0, 1, 2, or 3 mismatches to the A2 guide strand sequence. The accompanying table lists genes with fully complementary off-target sites. (**B**) Schematic representation of the guide strands of the designed shRNAs. (**C**) Table presenting the number of predicted off-target transcripts with 0, 1, or 2 mismatches to A2 and the newly designed guide strands, based on NCBI BLAST analysis. (**D**) Graphs showing luciferase reporter knockdown efficiency of shRNAs tested using two constructs containing either 16 or 85 CAG repeats, representing normal and mutant HTT alleles, respectively. Dots represent the mean firefly-to-Renilla luciferase ratio (F/R) ± SEM from three biological replicates, each with three technical replicates. Asterisks indicate statistically significant differences between CAG repeat lengths at 500 ng of amiRNAs, as determined by two-way ANOVA with Sidak’s correction. (**E**) Western blot analysis of HTT protein levels in HD fibroblasts (GM04281, 68/17Q) at 7 days post-transduction with lentiviral particles containing shRNAs (MOI = 10). Protein band intensities were normalized to plectin and compared using a one-way ANOVA with Tukey’s correction. Bars represent mean protein levels ± SEM from three biological and at least three technical replicates. *P*-values are indicated by asterisks (*P < .05, **P < .01, ***P < .001, ****P < .0001).

In our previous study, we demonstrated that amiR136-A2, composed of pri-miR136 scaffold and A2 insert (5′-CTGCTGCAGCTGCTGCTGC-3′), selectively silences mutant huntingtin (mutHTT) in cellular models of HD and in HD mice [26]. This molecule contains an interruption within the CTG sequence at position 8 from the 5′ end, forming a single A:A mismatch with the target CAG tract in the transcript. The mismatch is essential for the preferential silencing of mutated alleles through the translation inhibition mechanism. Although amiR136-A2 was well-tolerated in HD mice for up to 20 weeks, it still poses a risk of nonspecific binding to CAG-rich sequences in the human genome. Therefore, we conducted bioinformatic mapping of A2 across the human genome to identify its optimal modifications to minimize the risk of off-target effects. The analysis revealed 10 loci that showed full sequence complementarity to A2 (Fig. 1A). In the case of three genes (*TOX3, PEG3, MINK1*), full complementarity occurred simultaneously in the coding region (Consensus Coding Sequence, CCDS) and in the UTR, which may further increase the risk of off-target activity. However, most potential off-target sequences were only partially complementary to A2 (Fig. 1A and Supplementary Fig. S1A). Then, we examined how an additional mismatch at each position of the A2 molecule would affect the number of potential off-targets (Supplementary Fig. S1B–D). We observed that a mismatch at nucleotide positions 9, 11, and 13 of A2 significantly reduces the number of complementary sequences in the human genome. Based on this information and the literature [27], we designed a set of guide strands, including 9A (G > A), 10A (C > A), 11A (U > A), 13G (C > G), and 13A (C > A) (Fig. 1B). Compared to A2, these new molecules have a significantly lower number of complementary sequences in the human transcriptome, which we confirmed using the NCBI BLAST tool (Fig. 1C). Among all tested variants, molecule 13A showed the lowest number of off-target sequences, both with full complementarity and mismatches, making it the most promising candidate for further functional analysis.

Then, we conducted a luciferase reporter assay to assess the efficacy and allele selectivity of the newly designed molecules. HEK293T cells were co-transfected with constructs carrying the shRNAs and plasmids encoding exon 1 of the *HTT* gene with 16 or 85 CAG repeats, representing the normal and mutant alleles, respectively. All molecules reduced the level of mutHTT, while the effectiveness and selectivity differed depending on the position of the introduced substitution (Fig. 1D). Among the tested molecules, sh11A, sh13G, and sh13A demonstrated robust selectivity and efficiency in target knockdown and were selected for further validation in HD patient-derived fibroblasts. Western blot analysis confirmed that sh11A, sh13G, and sh13A significantly reduced the mutHTT protein level, achieving up to 75% silencing in the case of sh13G (Fig. 1E).

These results indicate that introducing an additional substitution in the A2 molecule does not reduce its effectiveness. Due to the similar activity profiles of the selected variants, we decided to re-evaluate them in the amiRNA format.

### CAG-targeting amiRNAs lower mutant huntingtin, ataxin 1, ataxin 3, and atrophin 1 levels in HD, SCA1, SCA3, and DRPLA fibroblasts

**Figure 2.**
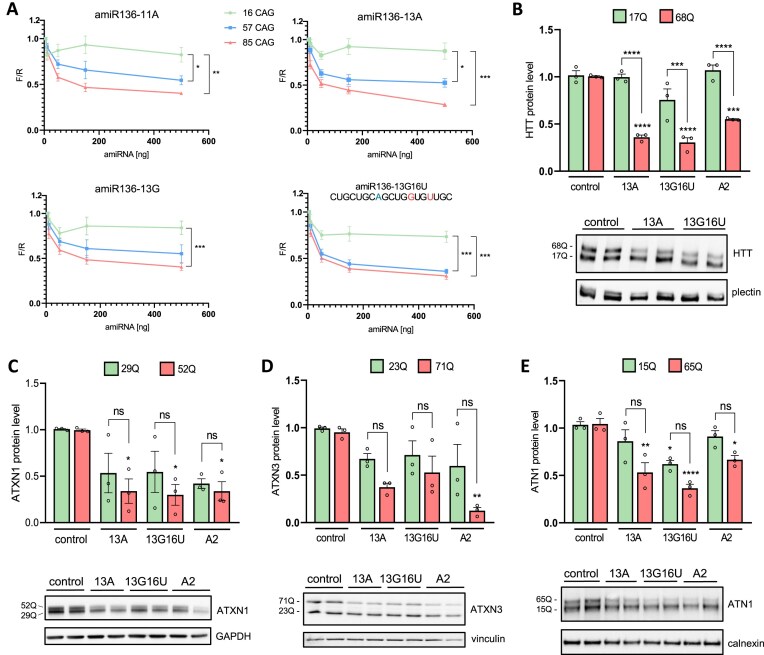
Analysis of amiRNAs activity in different models of polyQ diseases. (**A**) Graphs show the knockdown efficiency of luciferase reporters by amiRNAs using three constructs containing 16, 57, and 85 CAG repeats in exon 1 of the HTT gene. Dots represent mean firefly-to-Renilla luciferase ratios (F/R) ± SEM from three biological replicates, each with three technical replicates. Asterisks indicate statistically significant differences between CAG repeat lengths at 500 ng of amiRNA, as determined by two-way ANOVA with Sidak’s correction. Western blot analysis of polyglutamine protein levels in fibroblasts derived from patients with (**B**) HD (GM04281). The data for amiR136-A2 shown in the graph are derived from a previous study [26], (**C**) spinocerebellar ataxia type 1 (SCA1; GM06927), (**D**) spinocerebellar ataxia type 3 (SCA3; GM06153), and (**E**) DRPLA (GM13717), 7 days post-transduction with lentiviral particles expressing amiRNAs (MOI = 10). Protein band intensities were normalized to plectin, GAPDH, vinculin, and calnexin, respectively, and compared using one-way ANOVA with Tukey’s post hoc correction. Bars represent mean normalized protein levels ± SEM from three biological replicates and at least three technical replicates. *P*-values are indicated by asterisks (*P < .05, **P < .01, ***P < .001, ****P < .0001).

Selected siRNA sequences were embedded within the human pri-miR-136 scaffold, which was selected based on our previous study [26]. The efficacy of amiRNAs was determined using a luciferase reporter assay with target plasmids containing 16 CAG, 57 CAG, and 85 CAG repeats in exon 1 of the *HTT* gene. All molecules demonstrated effective and allele-selective silencing of *mutHTT* (Fig. 2A). AmiR136-13A exhibited the highest silencing efficiency for the mutant variants (57 CAG and 85 CAG), while amiR136-11A showed the lowest silencing activity (Fig. 2A). During the cloning process, we unintentionally generated a construct containing an additional, third interruption in the CAG tract at position 16 (C > T substitution). Using this construct, we obtained 60% silencing of the mutant allele and 15% silencing of the normal allele. The MTT assay revealed no significant cytotoxicity of the tested amiRNAs in HEK293 cells (Supplementary Fig. S2). Based on silencing efficacy and allele selectivity, amiR136-13A and amiR136-13G16U were selected for further studies in HD fibroblasts (Fig. 2B). In addition, we tested the versatility of amiRNAs in lowering other polyQ proteins, including ataxin 1 (SCA1), ataxin 3 (SCA3), and atrophin 1 (DRPLA) (Fig. 2C–E). The cells were transduced with lentiviral particles containing amiRNA cassettes, and protein levels were analyzed by western blotting. Although both amiRNAs effectively reduced polyQ protein levels by ≥50%, statistically significant allele-selectivity was observed only for the HD model (Fig. 2B). AmiR136-13A demonstrated the most favorable silencing pattern across polyQ disease models among the tested molecules. Given this finding and the fact that amiR136-13A has the most favored off-target profile, it was selected for further study.

### amiR136-13A is efficiently processed in HEK293T cells

**Figure 3.**
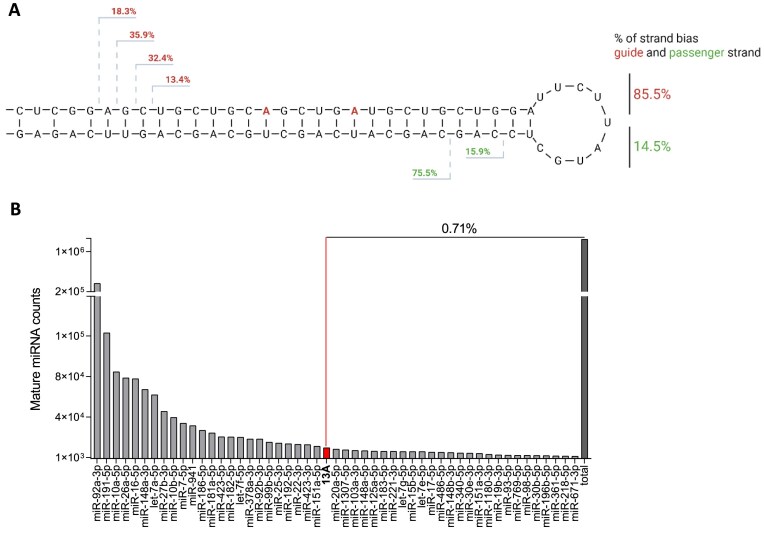
Next-generation sequencing of small RNAs in HEK293T cells following amiR136-13A transfection. (**A**) The most frequently released variants of the 13A guide and passenger strands are grouped according to their 5′ start sites. The guide strand is shown in red, and the passenger strand in green. Cleavage sites are indicated by dashed lines. Percentages represent the relative abundance of each variant. The overall strand bias is shown on the right. (**B**) Read counts of the 50 most highly expressed mature miRNAs after transfection. Total read counts for all 13A guide strand variants are included. The relative abundance of 13A among the top 50 expressed miRNAs is indicated.

It is well established that endonucleases Drosha and Dicer usually generate a heterogeneous pool of RNA products. Therefore, an essential step in evaluating the safety of amiR136-13A was to assess its processing efficiency and quality by the endogenous RNAi machinery. HEK293T cells were transfected with a plasmid encoding amiR136-13A, and small RNA sequencing was performed. First, we determined the relative proportion of guide and passenger strand variants released from amiR136-13A (Fig. 3A). A marked predominance of the guide strand derived from the 5′ arm (85.5%) over the passenger strand (14.5%) was observed. The predominant variants carried mismatches at positions 9 and 14, comprising 35.9% of the pool, while those with mismatches at positions 8 and 13 represented 32.4%. The released RNA molecules were also characterized by heterogeneity at their 3′ end and, consequently, varied in length, ranging from 19 to 23 nucleotides (nt). Analysis of the 50 most highly expressed endogenous miRNAs in the cellular model revealed that the mature 13A molecule accounted for only 0.71% of the total miRNA pool (Fig. 3B). This relatively low abundance indicates that amiR136-13A does not compete with endogenous miRNAs for RNAi pathway components and is unlikely to disrupt their expression.

### amiR136-13A efficiently reduces mutHTT level *in vivo* at half the dose of amiR136-A2

**Figure 4.**
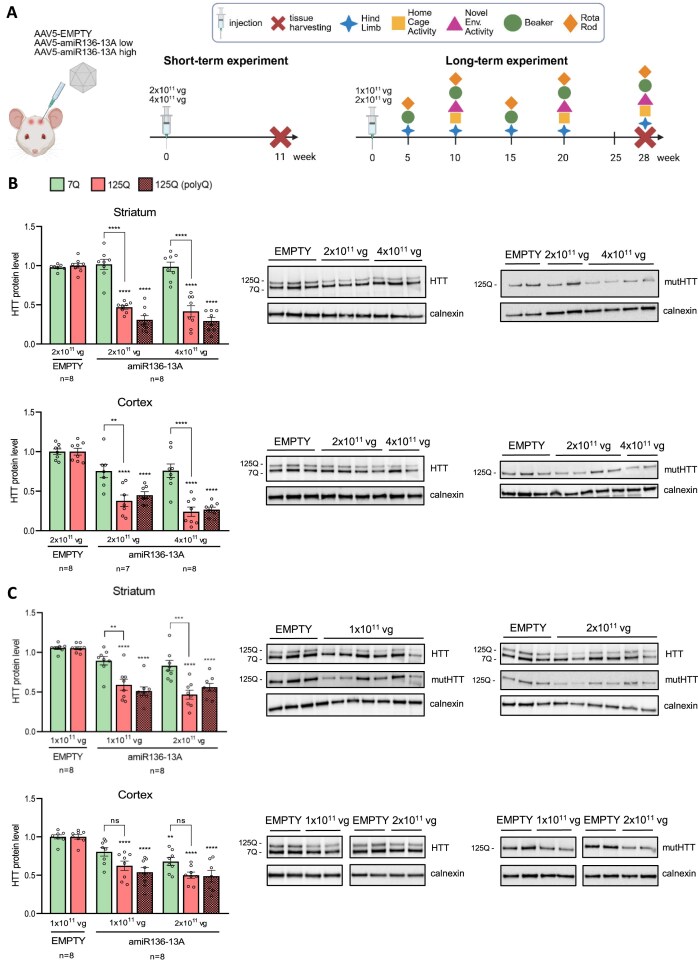
Safety and efficacy of amiR136-13A in YAC128 mice. (**A**) Study design illustrating the timeline of short-term and long-term experiments, along with the variants of the injected vector. (**B, C**) Western blot analysis of HTT protein levels in the striatum and frontal cortex at 11 and 28 weeks post-injection, respectively. Bars represent mean protein levels ± SEM, calculated from seven or eight biological replicates and at least three technical replicates. Mutant HTT protein levels are shown in red, while normal HTT levels are shown in green. Protein band intensities were normalized to calnexin. Statistical analysis was performed using one-way ANOVA followed by Tukey’s post hoc test. *P*-values are indicated by asterisks (**P < .01; ***P < .001; ****P < .0001). ns—not statistically significant; *n*—number of brain hemispheres.

To assess the *in vivo* efficiency and safety of amiR136-13A, we employed the YAC128 transgenic mouse model of HD. These animals carry the full-length human *HTT* gene with 125 CAG repeats, interrupted by nine CAA repeats, in addition to two normal murine alleles, which enables evaluation of allele-selective silencing [41]. The study consisted of a short-term (11-week) pilot phase to optimize dosing and a long-term step (28 weeks) to assess amiRNA safety. In the pilot experiment, mice (*n* = 4 per experimental group) were bilaterally injected into the striatum with the AAV5 vector carrying amiRNA at two doses: low (2 × 10^11 vg per hemisphere) and high (4 × 10^11 vg per hemisphere). Control animals received an equivalent low dose of the empty AAV5 vector (Fig. 4A). First, we analyzed the HTT level in three brain regions most affected in HD: the striatum, hippocampus, and frontal cortex. Two antibodies were used: one recognizing full-length HTT (human transgene and mouse wt Htt), and another specific to the polyQ tract, which is present only in the mutant form (human transgene). In the striatum and cortex, amiR136-13A treatment resulted in robust silencing of mutHTT, with approximately a 70% reduction in protein levels at the higher dose (Fig. 4B). Similar silencing was observed in the hippocampus (Supplementary Fig. S3A). Importantly, wt Htt levels remained unchanged in the striatum and cerebral cortex, regardless of the administered dose (Fig. 4B).

Given the strong mutHTT silencing efficiency observed, we proceeded with a follow-up long-term study using a reduced vector dose (half of that used in the initial experiment), corresponding to half the dose previously tested for amiR136-A2 [26]. Notably, even at reduced dosage (1 × 10^11 vg and 2 × 10^11 vg), amiR136-13A achieved ~50% silencing of mutHTT in the striatum and cortex (Fig. 4C), with comparable effects observed in the hippocampus (Supplementary Fig. S3B). A reduction (~30%) of wt Htt in the cortex was observed in high-dose-treated mice. It is worth noting that in our previous study [26], a 50% reduction in mutHTT levels in the striatum was observed only at the high dose (4 × 10^11 vg per hemisphere), and this level was not achieved in the cortex at any dose (Supplementary Fig. S3C).

In summary, amiR136-13A demonstrated sustained and robust efficiency in lowering mutHTT protein levels across all examined brain regions, even at a reduced dose. This effect persisted for at least 28 weeks following vector administration, further highlighting the therapeutic potential of amiR136-13A. Despite loss of allele specificity in the cortex, amiR136-13A still shows a preference for a longer CAG repeat tract.

### amiR136-13A is well tolerated *in vivo* and does not induce side effects

Throughout the experiments, animals were regularly monitored for changes in body weight and behavioral abnormalities. Consistent with previously published reports [42, 43], untreated male YAC128 mice exhibited increased body weight compared to WT controls (Supplementary Fig. S4A). This trend was not observed in females, where body weight varied across groups, limiting interpretation (Supplementary Fig. S4B). There were no statistically significant differences between groups, and treated animals (both doses) maintained stable body weight until 28 weeks post-injection (Supplementary Fig. S4C). The weight of selected organs did not differ between the control group and amiR136-13A-treated animals (Supplementary Fig. S5A–D). Biochemical analysis of mouse serum 20 weeks after surgery revealed no significant differences between the experimental groups (Supplementary Fig. S6). No alarming changes in animal behavior were observed, and behavioral tests confirmed previous reports of a mild phenotype in YAC128 mice [16]. We did not observe significant differences between control mice and amiRNA-treated animals using an extensive battery of tests, including the rotarod, home cage activity, hind limb clasping, beaker test, and novel environment activity analysis (Supplementary Fig. S7A–E).

To assess the long-term safety of amiR136-13A, we examined astroglial (glial fibrillary acidic protein, *Gfap*) and microglial (ionized calcium-binding adapter molecule 1, *Iba1*) markers. RT-qPCR analysis revealed no statistically significant changes in their expression levels, indicating that the amiRNA and the AAV5 vector were well-tolerated (Supplementary Fig. S8A and B). In addition, we evaluated serum levels of neurofilament light chain (NfL), a biomarker of neuronal damage, which was reported to be elevated in plasma and CSF of HD patients and in animal models [44, 45]. As expected, YAC128 mice exhibited significantly elevated NfL concentrations compared to WT controls, confirming the presence of neurodegenerative changes in this model. Treatment with amiR136-13A did not lead to any significant changes in NfL levels (Supplementary Fig. S8C). Furthermore, no correlation was found between mutHTT levels in the brain and NfL concentrations in the serum (Supplementary Fig. S8C). To further investigate systemic inflammation, spleens from YAC128 mice were analyzed by immunophenotyping (Supplementary Table S5). No significant differences were observed between the experimental groups in immune cell populations or cytokine levels. (Supplementary Figs S9 and S10). These results confirm that amiR136-13A, delivered via AAV5, does not exacerbate neurodegeneration or induce a systemic inflammatory response.

Finally, we analyzed the potential side effects caused by the overexpression of amiR136-13A. First, we characterized the quantity and quality of products resulting from amiR136-13A processing *in vivo* using small RNA sequencing. We confirmed a significant predominance of the guide strand (94.4%), which was even higher than in HEK293T cells (Supplementary Fig. S11A). In addition, greater homogeneity of the 5′ end was observed in the striatum, and only two main variants of the guide strand with mismatches at positions 9 and 14 (76%) and positions 8 and 13 (19%) were detected. Similar to the HEK293T model, the level of mature small RNAs derived from amiR136-13A was very low. In the striatum, it represented only 0.02% of the level of the 50 most abundant miRNAs (Supplementary Fig. S11B). In addition, administration of amiR136-13A induced only mild changes in the endogenous miRNA levels compared to the empty vector (Supplementary Fig. S11C). NCBI BLAST analysis identified nine potential guide strand binding sites (off-targets) that have one mismatch to 13A in the mouse transcriptome. Analysis of the expression level of these genes using RT-qPCR did not show any significant changes compared to the control, confirming the high specificity of the amiR136-13A molecule (Supplementary Fig. S11D).

### amiR136-13A does not induce global transcriptomic changes in NSCs

Due to differences in the mouse and human genomes, the specificity of therapeutic candidates should be analyzed in human cells. To evaluate potential changes in gene expression, including possible sequence-dependent off-target effects, we performed mRNA sequencing of HD NSCs after lentiviral transduction of amiR136-13A. HD iPSCs (ND42222) were differentiated into NSCs using a monolayer culture protocol. To verify the proper course of differentiation, we assessed the expression of NSC-specific markers (*SOX1, SOX2, PAX6*) and the pluripotency marker (*OCT4*) and confirmed acquisition of the appropriate phenotype (Supplementary Fig. S12A and B). We then confirmed *HTT* silencing after transduction of NSCs with the amiR136-13A lentiviral vector. Western blot analysis demonstrated an ~40% reduction in mutHTT level, confirmed with two different antibodies (Supplementary Fig. S12C). We did not observe any significant differences in the levels of other polyQ proteins, including ATN1, ATXN1, ATXN3, ATXN7, and the androgen receptor (AR), confirming the selectivity of amiR136-13A toward expanded CAG tracts (Supplementary Fig. S12D).

**Figure 5.**
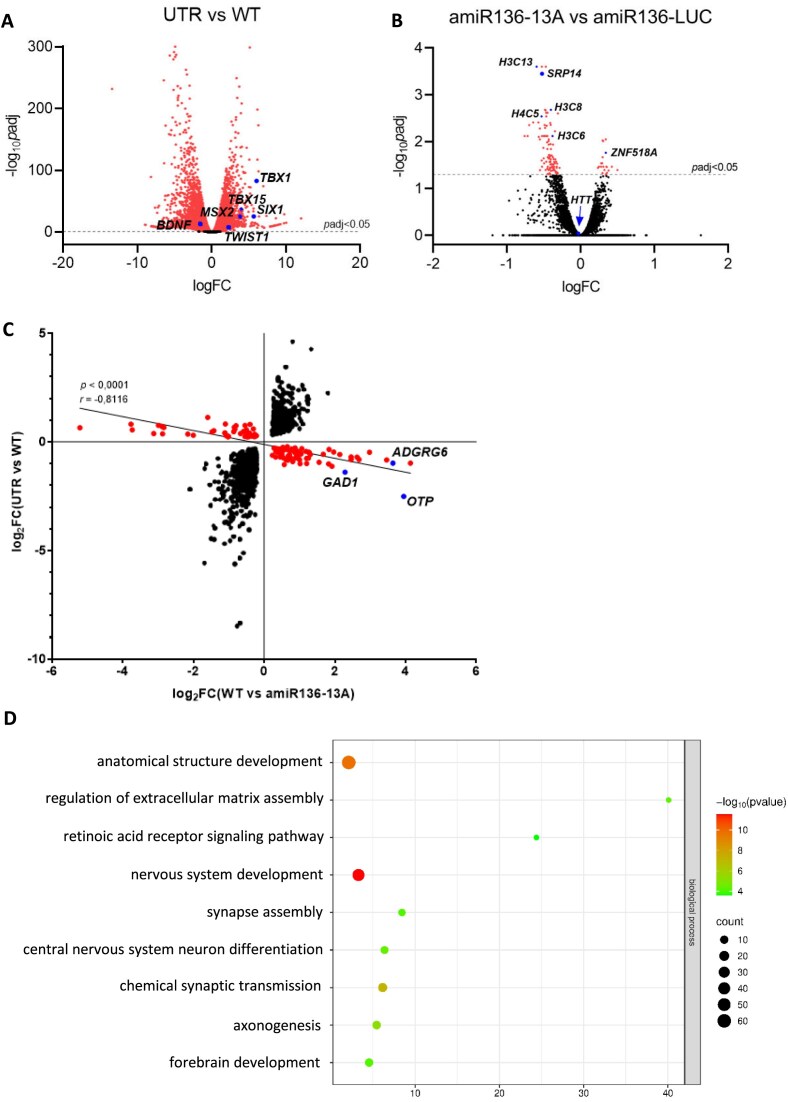
Transcriptomic changes in NSC HD after administration of amiR136-13A. Differential gene expression (DEG) analysis between (**A**) untreated HD NSCs (UTR) and healthy isogenic control cells (WT), and (**B**) HD NSCs transduced with lentiviruses carrying amiR136-13A and HD NSCs transduced with lentiviruses carrying amiR136-LUC (control). The *X*-axis shows the log₂ fold change (logFC) in gene expression, while the *Y*-axis shows the negative base-10 logarithm of the adjusted *P*-value [−log_10_(*p*adj)]. Genes significantly altered (*P* < .05) are highlighted in red. (**C**) Correlation between mRNAs that were significantly changed in UTR HD NSCs and those that were significantly changed after amiR136-13A treatment. The *X*-axis shows the log₂ fold change (log_2_FC) of significantly altered genes (*P* < .05) in NSCs treated with amiR136-13A compared to WT NSCs. The *Y*-axis shows the log₂FC for the same genes in untreated HD NSCs relative to WT NSCs. Each dot represents one gene that was significantly altered in both comparisons. Genes highlighted in red exhibited an opposite direction of change in the two comparisons, indicating that amiR136-13A treatment shifted their expression toward levels observed in healthy WT cells. The diagonal line represents the Pearson correlation between expression changes (*r* = –0.8116, *P* < .0001). (**D**) Bubble plot representing Gene Ontology (GO) of deregulated genes whose expression after amiR136-13A treatment approached the levels observed in WT NSCs.

Next, we performed RNA sequencing on HD NSCs transduced with amiR136-13A or amiR136-LUC (negative control), untransduced HD NSCs, and healthy isogenic WT control cells. PCA and sample divergence analysis demonstrated proper clustering of samples (Supplementary Fig. S13A and B). Differential gene expression (DEG) analysis comparing untreated HD NSCs to WT controls revealed characteristic features of the HD NSC transcriptome, including deregulation of transcription factors such as *TBX1, SIX1, TWIST1, MSX2*, and *TBX15*, consistent with previous findings (Fig. 5A and Supplementary Fig. S14A) [3].

Comparison of HD NSCs transduced with amiR136-13A versus amiR136-LUC revealed only minor changes in the gene expression profile (Fig. 5B). Statistically significant changes were observed for 122 genes, of which 102 were downregulated and 20 were upregulated. Most of these genes do not have a sequence complementary to 13A. GO analysis revealed that the deregulated genes are primarily associated with chromatin remodeling and organization processes (i.e. *H3C13, H3C8*) (Supplementary Fig. S14B). As expected, the level of *HTT* mRNA did not change significantly. Interestingly, we observed a modest reduction in the expression of the signal recognition particle 14 gene (S*RP14*). This gene displays partial complementarity to the 13A guide strand sequence, containing two mismatches and eight CAG repeats. This finding was further evaluated using RT-qPCR and western blot analysis, which showed no change in *SRP14* expression levels (Supplementary Fig. S15A and B).

To investigate the impact of lentiviral transduction on gene expression, we performed DEG analysis between HD NSCs transduced with amiR136-LUC and untreated HD NSCs. This comparison revealed substantial transcriptomic changes, involving 810 genes with significantly altered expression (Supplementary Fig. S16A). GO analysis indicated that the deregulated genes are involved in numerous biological processes, including neurogenesis, angiogenesis (*PDGFRA, NRP1*), VEGF signaling (*VEGFA*), and developmental processes (*PTCHD4*) (Supplementary Fig. S16B). These findings demonstrate that the lentiviral transduction process itself can lead to considerable transcriptomic alterations.

To assess the potential effect of restoring a normal gene expression profile following amiR136-13A treatment, we compared the common pool of significantly altered genes identified in both the untreated versus WT NSCs comparison and the amiR136-13A-treated versus WT NSCs comparison. A strong and statistically significant inverse correlation was observed (*r* = –0.8116; *P* < .0001), indicating that the introduction of amiR136-13A into HD NSCs partially “reversed” the deregulation profile of 111 genes toward expression levels typical of healthy cells (Fig. 5C). Interestingly, GO analysis of the genes whose expression was restored by amiR136-13A toward levels observed in healthy cells revealed significant enrichment of biological processes related to nervous system development (i.e. *OTP, GAD1, ADGRG6*). In particular, processes such as “anatomical structure development of the nervous system,” “synapse formation,” “neuronal differentiation,” and “chemical synaptic transmission” were prominently represented (Fig. 5D).

These findings suggest that amiR136-13A is a safe molecule, exhibiting no evidence of global transcriptomic disruption or activation of undesired cellular pathways in the studied models.

### amiR136-13A reduces pathogenic HTT1a protein level

**Figure 6.**
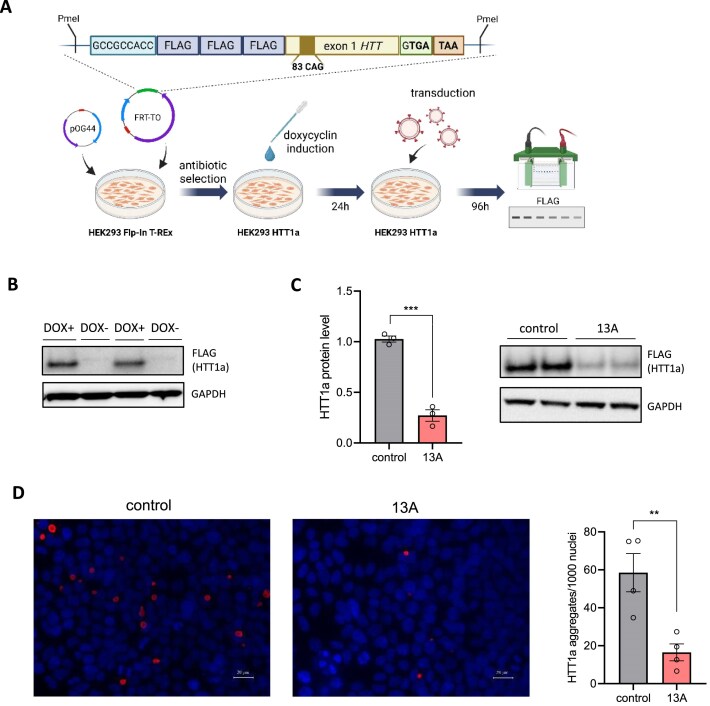
Analysis of HTT1a silencing. (**A**) Experiment workflow. (**B**) A representative western blot analysis confirming the inducible expression of the *HTT1a* transgene. (**C**) Western blot analysis of HTT1a level. Protein band intensities were normalized to GAPDH and compared using a Student’s *t*-test. amiR136-Luc was used as a control. Bars on the graph represent mean protein levels ± SEMs from three biological and three technical replicates. (**D**) Immunohistochemical staining using anti-FLAG antibody to show HTT1a aggregates (red spots). The bars on the graph show the number of HTT1a aggregates per 1000 nuclei after amiR136-13A treatment ± SEMs from four biological and three technical replicates. Scale bar = 20 µm. *P-*values are indicated by asterisks (*P < .05; **P < .01; ***P < .001).

Previous studies suggest that the level of the toxic HTT1a protein increases with patient age, and it may play a significant role in the pathogenesis of HD [46, 47]. Therefore, therapeutic solutions are being sought to target the *HTT1a* transcript alone or in combination with full-length mutHTT. Because CAG repeat-targeting molecules, in theory, should inhibit translation of both protein variants, we tested this hypothesis using amiR136-13A. Due to the difficulties in detecting the endogenous form of HTT1a protein, we designed a simplified construct based on the inducible Flp-In T-REx -293 system, which enables controlled overexpression of the truncated protein (Fig. 6A). To facilitate reliable detection of the resulting protein, an N-terminal 3xFLAG epitope tag was introduced. First, we confirmed that the doxycycline induction results in the formation of HTT1a protein (Fig. 6B). Subsequently, cells were transduced with lentiviral vectors encoding amiR136-13A to assess *HTT1a* silencing. A marked reduction in HTT1a protein level was observed, reaching up to 75% compared to the control (Fig. 6C). In addition, we observed a significant reduction in the number of HTT1a aggregates in cells treated with amiR136-13A (Fig. 6D and Supplementary Fig. S17). The obtained results confirm that amiR136-13A is also effective against the truncated, pathogenic form of the HTT protein, further supporting its therapeutic potential.

## Discussion

As our understanding of disease mechanisms improves, therapeutic approaches must be continuously refined. The strategy of targeting CAG repeats has traditionally been viewed as risky due to the possible impact on other repeat-containing regions in the human genome. Here, we demonstrated that a carefully optimized amiRNA molecule is safe and efficient in silencing of mutHTT in cellular and mouse models of HD.

Designing allele-selective, repeat-targeting small RNAs is challenging, as any sequence modification carries the risk of losing activity or selectivity. The position and type of mismatch with the target mRNA play a key role in maintaining this delicate balance. The most effective canonical miRNA target sites include a perfect match to the seed region (nucleotides 2–8) [48, 49], which is further supported by supplementary pairing with the 3′ region of the miRNA (nucleotides 13–16) [50]. Therefore, the design of CAG-targeting molecules acting as miRNA allows the introduction of mismatches in their central region [51, 52]. Surprisingly, in our study, the best-performing amiR136-13A releases two dominant variants that contain a second mismatch in the 3′ region of the molecule (position 13 or 14). This further supports the unique nature of these miRNA-like small RNAs, which inhibit translation of long CAG tracts by cooperatively binding multiple RISCs within the coding region of the gene.

AmiRNA design is also challenging due to difficulties in predicting the outcome of cellular processing [53–55]. We observed slight differences in the proportions of the main variants released from amiR136-13A in HEK293T cells and mouse striatum. Since the quality of processing may influence the on- and off-target activity of small RNAs, it would be interesting to evaluate it in other brain regions and human neurons.

The results of small RNA sequencing from different models are consistent in terms of the very low level of CAG-targeting small RNAs released from the pri-miR136 backbone, resembling those of endogenous miRNA [26, 56]. It reduces the risk of non-specific binding while providing ≥50% reduction in mutHTT, which is considered therapeutically beneficial. Indeed, we confirmed that amiR136-13A did not significantly influence the level of other transcripts in mouse striatum and human NSCs. It also did not change the level of other polyQ proteins in NSCs. In addition, we did not observe signs of RNAi pathway saturation, as endogenous miRNA levels in the mouse striatum were similar to those in controls. A detailed analysis of biochemical parameters, immune response markers, and mouse behavior showed no differences compared to control animals. Although analysis of serum NfL levels confirmed neuronal damage in YAC128 mice, amiRNA treatment did not result in a significant improvement. This is consistent with recent findings from the Hayden group, who quantified NfL levels in both serum and CSF of YAC128 mice following administration of an anti-HTT ASO [57]. Despite efficient silencing of mHTT expression in the brain, serum NfL levels remained unchanged. Given that NfL concentrations are markedly higher in CSF than in the blood of HD mouse models (YAC128, zQ175, R6/2) [58], CSF appears to represent a more sensitive matrix for detecting neurodegenerative changes, particularly in studies with limited sample sizes.

It is worth noting that the newly developed amiR136-13A effectively reduced mutHTT levels in various brain structures at a vector dose half that of amiR136-A2, significantly reducing the risk of non-specific effects associated with AAV5 transduction. Although AAV5 vectors are commonly used for amiRNA delivery to the brain due to their low immunogenicity, transduction of neurons and astrocytes, and retrograde axonal transport [59], their administration in clinical settings still requires further optimization. Similarly, any potential delayed toxicity arising from long-term expression of RNAi triggers will require thorough investigation in future studies.

In this study, we demonstrated for the first time that CAG-targeting amiRNA reduces the levels of both toxic forms of HTT, including full-length mutant HTT and HTT1a. Because, as a result of somatic expansion of CAG repeats, the proportion of HTT1a increases throughout the lifetime of an HD patient, reducing its level could have a beneficial therapeutic effect. Among the clinical trials currently underway, only those using non-allele-selective amiRNA (AMT-130) aim to reduce both forms of HTT (NCT04120493 and NCT05243017) [60]. Since we used an artificial model of HTT1a expression in our study, further research is needed to confirm the reduction of HTT1a in relevant models with endogenous *HTT* expression. Another limitation of our study is the lack of evidence of phenotypic improvement after amiR136-13A treatment. Similar to other studies, we did not observe significant differences between WT and YAC128 mice using a battery of tests, which suggests that this model is not the best choice for assessing behavioral changes [16, 61]. Moreover, although the YAC128 carries the full-length human *HTT* gene, the CAG repeat tract contains CAA interruptions, which are relevant when using a CAG-targeting strategy. In addition to its mild phenotype, the YAC128 model precludes reliable evaluation of allele selectivity, as the wild-type mouse allele contains only 6 CAG repeats interrupted by a CAA triplet, whereas the human gene typically contains ~15 pure CAG repeats. The rationale for using this mouse model was to directly compare the activity of our newly designed amiR136-13A with that of amiR136-A2, which had been previously evaluated in YAC128 [26].

A CAG-targeting strategy gives the unique opportunity to develop a universal therapeutic molecule for multiple polyQ diseases. Here, we show that amiR136-13A effectively reduces the levels of ataxin-1, ataxin-3, and atrophin-1, while maintaining a preference for the mutant protein. However, as demonstrated in this study and in previous reports [29, 30], achieving allele selectivity in these models is more challenging than in HD. This phenomenon may be partially explained by differences in the genomic localization of CAG repeats within individual genes, which may influence the translation inhibition mechanism. In the *HTT* gene, the CAG repeat is located at the 5′ end of the ORF (exon 1), in *ATN1* in the middle (exon 5), and in *ATXN3* near the 3′ end (exon 10). The observed lowering of multiple polyQ-expanded proteins supports the broader therapeutic potential of CAG-targeting amiRNAs, while the degree of selectivity may remain disease- and context-dependent.

In conclusion, our data show that amiR136-13A outperforms previously tested CAG-targeting amiRNAs in terms of efficacy, broader action in different brain regions, and a more favorable safety profile. These results confirm that the CAG-targeting strategy, utilizing AAV-amiRNA, offers a unique combination of features, including high efficacy, a preference for mutant alleles, and long-lasting expression after a single administration. It is also a promising solution for other polyQ diseases, for which clinical trials of gene therapies remain very limited.

## Supplementary Material

ugag023_Supplemental_File

## Data Availability

Raw small RNA-seq datasets were deposited in the National Center for Biotechnology Information (NCBI) Sequence Read Archive (SRA), with accession number PRJNA1347383. The raw total mRNA-seq dataset was deposited in the European Nucleotide Archive (ENA) with accession number PRJEB98143.
