# Supplementary material for: CAG-targeting artificial miRNA with reduced off-target risk for efficient lowering of pathogenic polyglutamine proteins
Source: NAR Mol Med. 2026 Apr 30;3(2):ugag023. doi: 10.1093/narmme/ugag023 (PMC13161563; doi:10.1093/narmme/ugag023)
Supplement: ugag023_Supplemental_File [file ugag023_supplemental_file.pdf]

Supplementary information

CAG-Targeting artificial miRNA With Reduced Off-Target Risk for Efficient Lowering of Pathogenic Polyglutamine Proteins

Marianna Pewinska-Kolodziejczak[1], Anna Kotowska-Zimmer[1], Lukasz Przybyl[2], Dorota Wronka[2], Anna Karlik[2], Michal Smuszkiewicz[1], Julia Balcerek[1], Joanna Suszynska-Zajczyk[3], Anna Piaszyk-Borychowska[1], Martyna Urbanek-Trzeciak[4], Gaurav Sablok[5], Emilia Kozłowska[6], Jan Podkowinski[5], Agnieszka Fiszer[6], Piotr Kozlowski[4], Luiza Handschuh[5] and Marta Olejniczak[1,*]

[1] Department of Genome Engineering, Institute of Bioorganic Chemistry, Polish Academy of Sciences, Noskowskiego 12/14, 61-704, Poznan, Poland
[2] Laboratory of Mammalian Model Organisms, Institute of Bioorganic Chemistry, Polish Academy of Sciences, Noskowskiego 12/14, 61-704, Poznan, Poland
[3] Department of Biochemistry and Biotechnology, Poznan University of Life Sciences, 60-632 Poznan, Poland
[4] Department of Molecular Genetics, Institute of Bioorganic Chemistry, Polish Academy of Sciences, Noskowskiego 12/14, 61-704, Poznan, Poland
[5] Laboratory of Genomics, Institute of Bioorganic Chemistry, Polish Academy of Sciences, Noskowskiego 12/14, 61-704, Poznan, Poland
[6] Department of Medical Biotechnology, Institute of Bioorganic Chemistry, Polish Academy of Sciences, Noskowskiego 12/14, 61-704, Poznan, Poland

Supplemental Table 1. Oligonucleotide sequences used to generate shRNA and amiRNA expression cassettes.

Name	Sequence 5′ → 3′
sh9A	GATCCGCAGCAGCAGCTGCAGCAGCTGCTTCCTGTCACAGCTGCTGCAACTGCTG CTGCTTTTTG
sh10A	GATCCGCAGCAGCAGCTGCAGCAGCTGCTTCCTGTCACAGCTGCTGCAGATGCTG CTGCTTTTTG
sh11A	GATCCGCAGCAGCAGCTGCAGCAGCTGCTTCCTGTCACAGCTGCTGCAGCAGCTG CTGCTTTTTG
sh13A	GATCCGCAGCAGCAGCTGCAGCAGCTGCTTCCTGTCACAGCTGCTGCAGCTGATG CTGCTTTTTG
sh13G	GATCCGCAGCAGCAGCTGCAGCAGCTGCTTCCTGTCACAGCTGCTGCAGCTGGTG CTGCTTTTTG
amiR136-11A	AATTCCACTCCACTGCCCGACGTCGCCTCGGTGGTGTTGGATGAGCCCTCGGAGC TGCTGCAGCAGCTGCTGCTGGATTCTTATGCTCCAGCAGCAGCTGCTGCAGCAGT TCAGAGGGTTCTATCATTTCGTCGGATGGAAAGGAGTGTATTCTGAAGATGC
amiR136-13A	AATTCCACTCCACTGCCCGACGTCGCCTCGGTGGTGTTGGATGAGCCCTCGGAGC TGCTGCAGCTGATGCTGCTGGATTCTTATGCTCCAGCAGCATCAGCTGCAGCAGT TCAGAGGGTTCTATCATTTCGTCGGATGGAAAGGAGTGTATTCTGAAGATGC
amiR136-13G	AATTCCACTCCACTGCCCGACGTCGCCTCGGTGGTGTTGGATGAGCCCTCGGAGC TGCTGCAGCTGGTGCTGCTGGATTCTTATGCTCCAGCAGCACCAGCTGCAGCAGT TCAGAGGGTTCTATCATTTCGTCGGATGGAAAGGAGTGTATTCTGAAGATGC

Supplemental Table 2. Sequences of primers used for RT-qPCR and genotyping.

Name	Forward 5′ → 3′	Reverse 5′ → 3′
mouse *β-actin*	ACTGTCGAGTCGCGTCCA	ATCCATGGCGAACTGGTGG
HTT	GTGCAGTGATGACGCAGAGT	TCTTCGGGTCTCTTGCTTGT
Htt	CCGCTCAGGTTCTGCTTTTA	TGGACAGGGAACAGTGTTGG
Ccdc177	TGCCAGCAGAAAAGACACAC	CGTCAGCTTTGTCTTCACCA
Arsb	TGGTGGACTCACATCGGTAA	ATAGCACTTCTTCGCCCTGA
Kmt2d	GCTCAACAGACATTGGCTCA	GCCAGTCAAGCCTCTTCAAC
Maml1	AGGCAGGGTTCCCTTACTGT	TTTGCCTCAGAACAGCCTTT
Mylip	GGCAGATGGGTGTTGAGAGT	GAGCATGTCCAGCACGTCTA
Tmem158	CAGGATCTGGTCACTTTCTGC	AAAGTGGGGTTTGTGCTGAG
Ppp1r3f	GCCGCATGACTAAAACCAAT	TGCTGCCTCTTCAATGTGTC
Tle1	GTGCCTCTTAGGCTGTCTGG	CCTCACCCTTCAGGACTTCA
Zc4h2	GAACCCAAGGCAAAAACAGA	TTACTGCCCCAGTTTGGAAC
Iba1	TCTGCCGTCCAAACTTGAAGCC	CTCTTCAGCTCTAGGTGGGTCT
Gfap	CACCTACAGGAAATTGCTGGAGG	CCACGATGTTCCTCTTGAGGT
SOX1	ACCAGGCCATGGATGAAG	CTTAATTGCTGGGGAATTGG
SOX2	CAAAAATGGCCATGCAGGTT	AGTTGGGATCGAACAAAAGCTATT
PAX6	TGCTCCGGCATGAAATATACTA	GTCTCCAAATGTGCAGCAAC
OCT4	AGTTTGTGCCAGGGTTTTTG	ACTTCACCTTCCCTCCAACC
EEF2	TCATCGAGGAGTCGGGAGAG	ACGACCGGGTCAGATTTCTTG

HTTex1	GCCGCCACCATGGACT	GGACTTGAGGGACTCGAAGG
SRP14	CCAAAGAAGGGTACTGTGGAGG	CCATGTTAGCTCTAAGGAGGTTTG

Supplemental Table 3. Antibodies used for western blotting and immunofluorescence.

Name	Supplier, catalog number	Dilution	Buffer
Primary antibodies			
Huntingtin	Abcam, ab109115	1:1000	5% milk in PBS-T
polyQ	Sigma-Aldrich, P1874	1:1000	5% milk in PBS-T
Androgen receptor	Santa Cruz Biotechnology, sc-7305	1:1000	5% milk in TBS-T
Ataxin-1	Thermo Fisher Scientific, 703273	1:1000	5% milk in PBS-T
Ataxin-3	Proteintech, 13505-1-AP	1:1000	5% milk in PBS-T
Ataxin-7	Thermo Fisher Scientific, PA1-749	1:1000	5% milk in TBS-T
Atrophin-1	Sigma-Aldrich, HPA031619	1:1000	5% BSA TBS-T
Vinculin	Cell Signaling, 4650S	1:1000	5% BSA TBS-T
Calnexin	Sigma-Aldrich, C4731	1:1000	5% milk in PBS-T
Plectin	Cell Signaling, 12254	1:1000	5% milk in TBS-T
FLAG-tag	Sigma-Aldrich, F1804	1:1000 (WB)<br>1:200 (IF)	5% milk in TBS-T (WB)<br>1% BSA in PBS (IF)
GAPDH	Millipore, MAB374	1:5000	5% milk in PBS-T
SRP14	Proteintech,, 11528-1-AP	1:1000	5% milk in PBS-T
Secondary antibodies			
M-POX	Jackson ImmunoResearch, 715-035-150	1:1000	Dependent on the primary antibody
R-POX	Jackson ImmunoResearch, 711-035-152	1:1000	Dependent on the primary antibody
M-POX	Jackson ImmunoResearch, 715-586-151	1:1000	1% BSA in PBS

Supplemental Table 4. The vector doses used in the *in vivo* experiments.

Experiment	amiRNA		Empty vector
	Low dose	*High dose*	
Short-term	$2x10^{11}$ vg per hemisphere	$4x10^{11}$ vg per hemisphere	$2x10^{11}$ vg per hemisphere
Long-term	$1x10^{11}$ vg per hemisphere	$2x10^{11}$ vg per hemisphere	$1x10^{11}$ vg per hemisphere

Supplemental Table 5. Antibodies used for immunophenotyping.

Antibody	Fluorochrome	Supplier	Dilution	Catalogue number
**Panel 1**				
CD3	V450	BD	1:20	561389
CD4	APC-vio770	Miltenyi Biotec	1:20	130-119-132
CD8	PerCP-Cy5.5	Thermo Fisher Scientific	1:100	45-0081-82
γδTCR	PE	BioLegend	1:50	107507
CD69	PE-Cy7	BioLegend	1:50	104512
CD62L	APC	Miltenyi Biotec	1:10	130-102-931
CD44	FITC	BioLegend	1:50	103005
**Panel 2**				
CD11b	PE	Miltenyi Biotec	1:150	130-113-806
CD11c	PerCP-Cy5.5	Thermo Fisher Scientific	1:50	45-0114-80
Gr-1	PE-Cy7	BioLegend	1:100	108415
Ly6C	APC-efluor780	Thermo Fisher Scientific	1:100	47-5932-80
B220	Alexa fluor647	BioLegend	1:100	103229
CD86	Efluor450	Thermo Fisher Scientific	1:25	48-0862-82
**Panel 3**				
CD4	APC-vio770	Miltenyi Biotec	1:20	130-119-132
CD25	BV711	Thermo Fisher Scientific	1:20	407-0251-82
FoxP3	PE-Cy5.5	Thermo Fisher Scientific	1:25	35-5773-82
RORγt	APC	Thermo Fisher Scientific	1:10	17-6988-82
Tbet	PE	Thermo Fisher Scientific	1:10	12-5825-82
Helios	Pacific blue	BioLegend	1:20	137220
Ki67	PE-Cy7	Thermo Fisher Scientific	1:50	25-5698-82
**Panel 4 (Restimulated)**				
CD3	V450	BD	1:20	561389
CD4	FITC	Thermo Fisher Scientific	1:50	11-0042-82
CD8	APC-vio770	Miltenyi Biotec	1:20	130-120-806
IL-17	PE	Miltenyi Biotec	1:15	130-112-009
IFNγ	PE-Cy7	BD	1:15	557649
TNFα	APC	Miltenyi Biotec	1:10	130-123-277
FoxP3	PE-Cy5.5	Thermo Fisher Scientific	1:25	35-5773-82

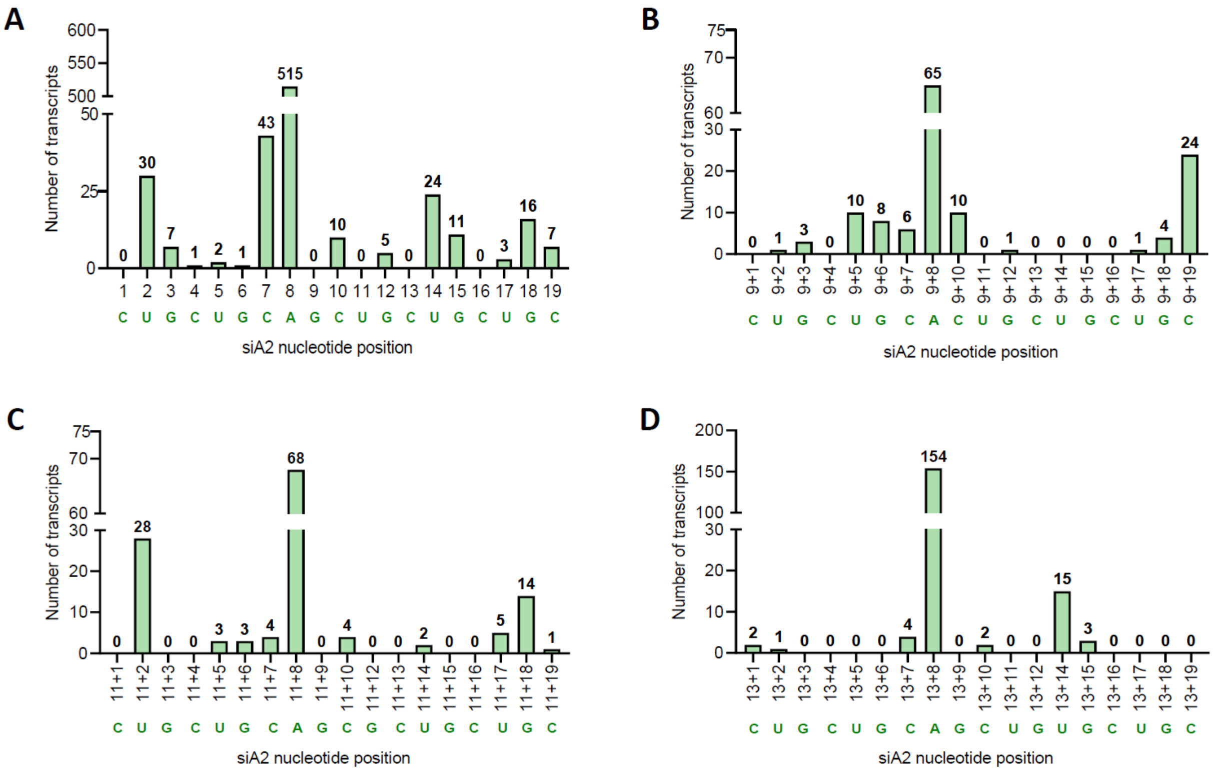

Supplementary Figure 1. Bioinformatic mapping of the A2 guide strand to the human genome using Bowtie (v.1.2.3). (A) Number of off-targets with a single mismatch at each specific nucleotide position of the A2 guide strand. (B-D) Number of off-targets with two mismatches, with one mismatch fixed at nucleotide position 9 (B), 11 (C), or 13 (D) of A2, respectively.

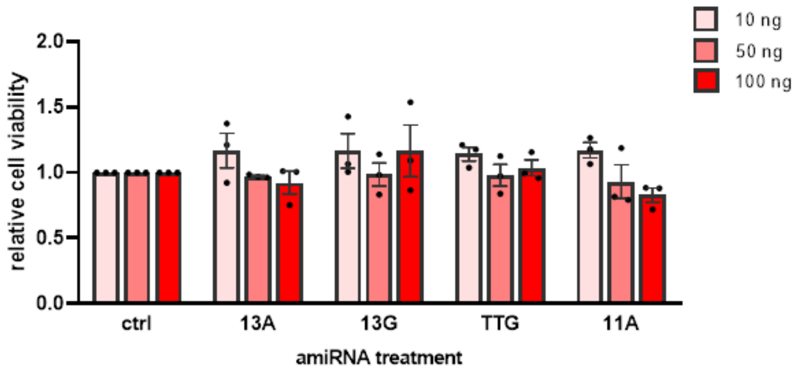

Supplementary Figure 2. Cell viability following transfection with plasmids encoding amiRNAs or an empty plasmid (ctrl). The MTT assay was performed 48 h after transfection of HEK293T cells with amiRNA-expressing plasmids at doses of 10, 50, or 100 ng. Signal intensities were normalized to the control. Statistical analysis was performed using two-way ANOVA. Bars represent mean viability ± SEM from three biological replicates.

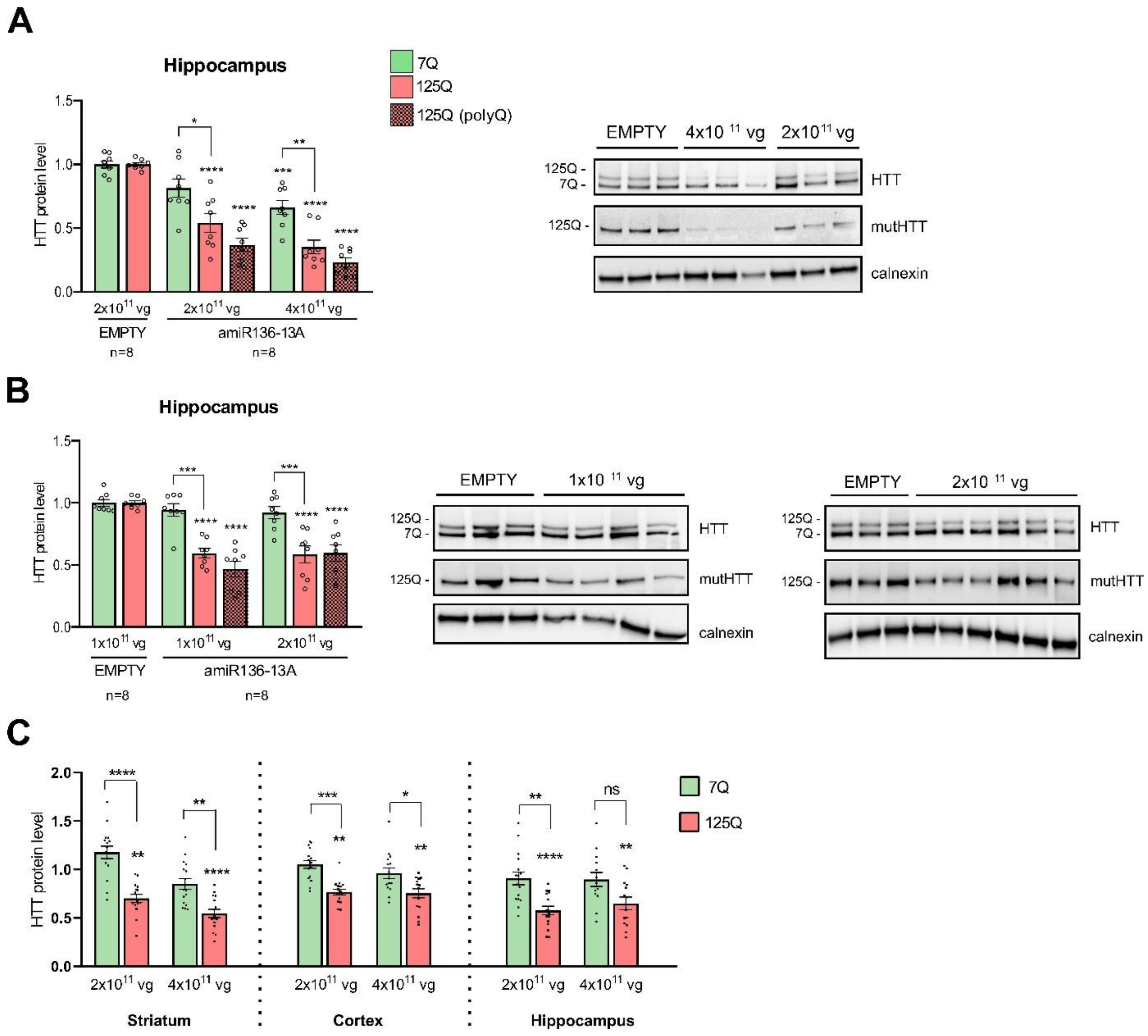

Supplementary Figure 3. Western blot analysis of HTT protein levels following intrastriatal injection of amiRNA in YAC128 mice. Analysis of the protein levels in the hippocampus at (A) 11 and (B) 28 weeks post-injection of amiR136-13A. Bars represent mean protein levels ± SEM, calculated from eight biological replicates (n=8) and at least three technical replicates. (C) HTT protein levels in different brain regions of YAC128 mice at 20 weeks post-injection of amiR136-A2. These results have been previously published (1) and are presented here for comparison. Bars represent mean protein levels ± SEM (n=16). Mutant HTT levels are shown in red, and wild-type HTT levels are shown in green. Protein band intensities were normalized to calnexin. Statistical analysis was performed using one-way ANOVA followed by Tukey's post hoc test. *p*-values are indicated by asterisks (**p* < 0.05; ***p* < 0.01; ****p* < 0.001; *****p* < 0.0001). n – number of brain hemispheres analyzed.

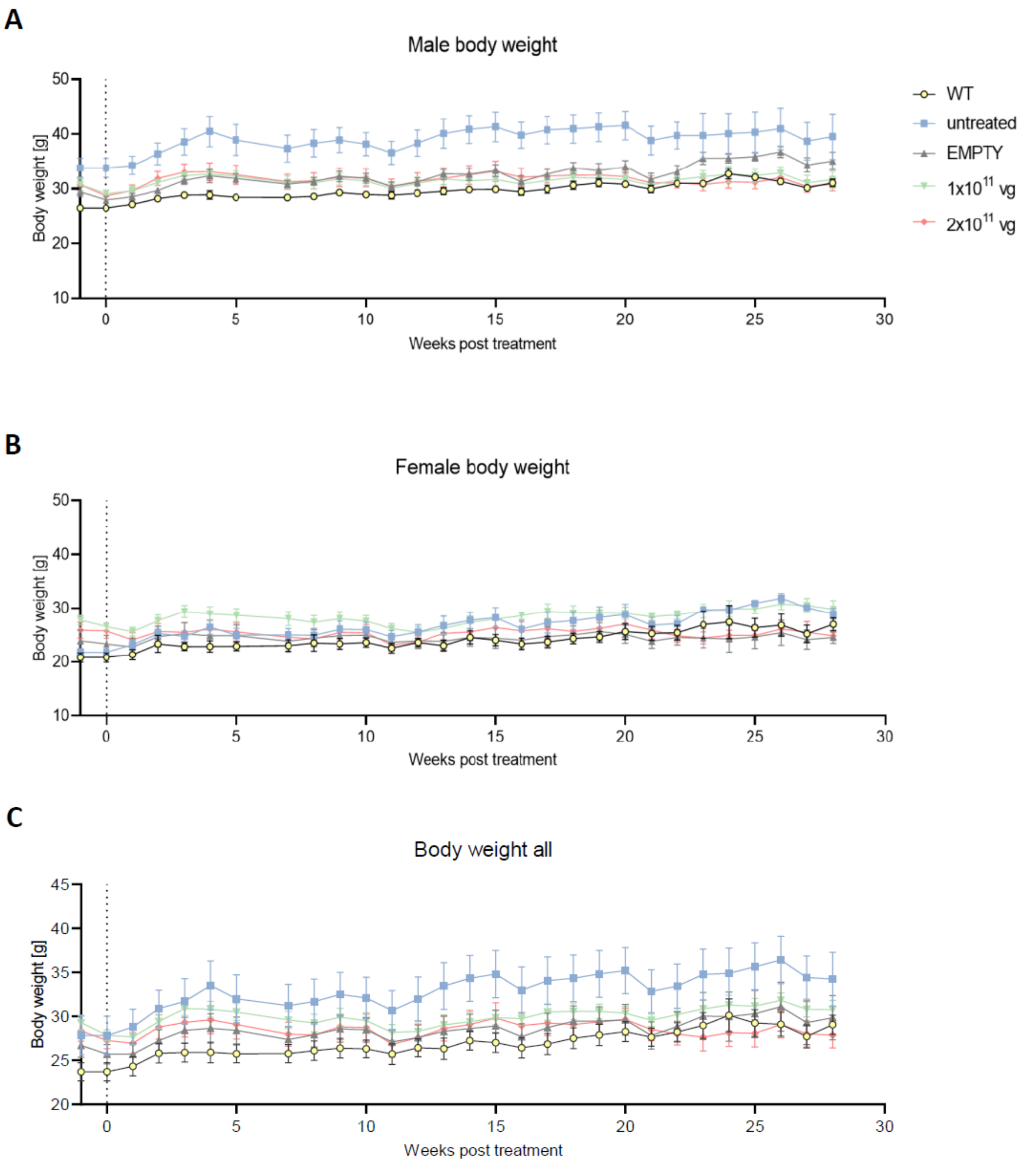

Supplementary Figure 4. Body weight changes over 28 weeks post-injection. (A) Mean body weight of male mice. (B) Mean body weight of female mice. (C) Mean body weight of all mice combined (both sexes). Body weight was measured weekly throughout the 28-week experimental period. Data points represent the mean ± SEM for each group at the indicated time point. The analysis included YAC128 mice treated with the AAV5 vector carrying amiR136-13A at two doses (1×10^{11} vg and 2×10^{11} vg), a control group receiving the empty vector, as well as reference groups of untreated YAC128 and wild-type (WT) mice. Statistical analysis was performed using two-way ANOVA followed by Tukey's post hoc test, which revealed no statistically significant differences between groups. For weeks 0-20, n=10 mice per group; for weeks 20-28, n = 6 mice per group.

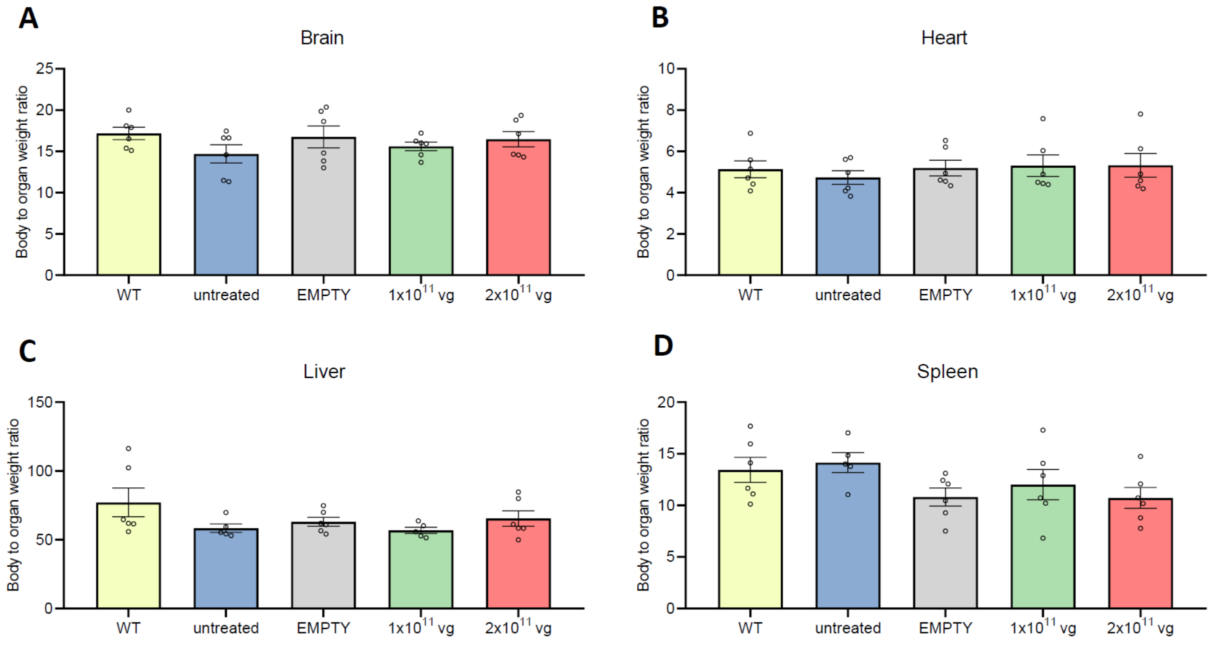

Supplementary Figure 5. Organ weights 28 weeks post-injection. Data represent n = 6 mice per group. Prior to analysis, data were screened for outliers and tested for normality using appropriate tests. For normally distributed data, a one-way ANOVA with Tukey's post hoc test was applied; for non-normally distributed data, a Kruskal-Wallis test with Dunn's multiple comparisons was used. Mean values are presented as ±SEM. Panels (A-D) show the ratio of organ weight to body weight, calculated using the formula: $\frac{organ\ mass\ [g]}{body\ weight\ [g]} * 1000$, for (A) brain; (B) heart; (C) liver; (D) spleen.

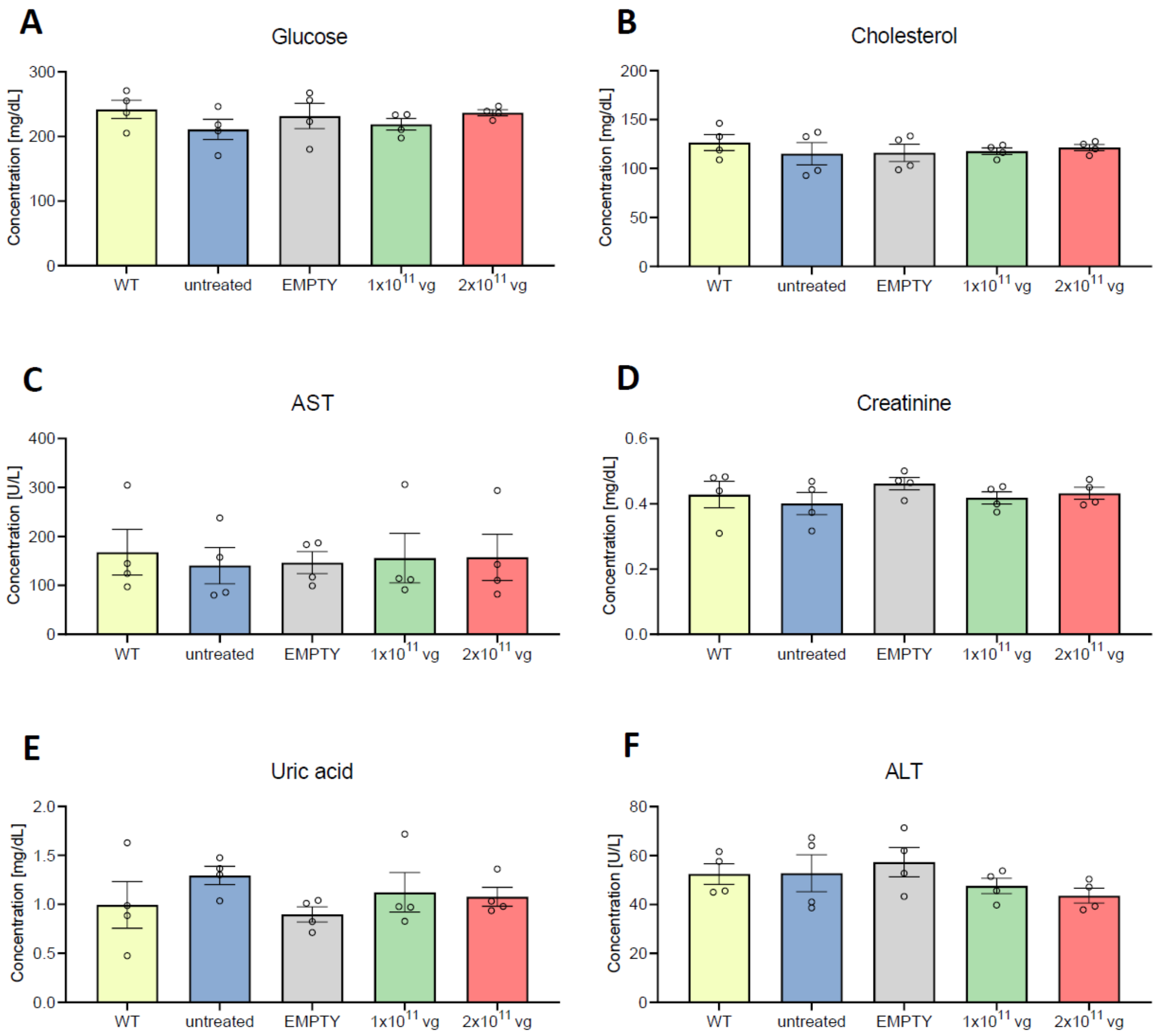

Supplementary Figure 6. Biochemical analysis of mouse serum 20 weeks post-surgery. Concentrations of each parameter are shown separately in panels: (A) glucose, (B) cholesterol, (C) aspartate aminotransferase (AST), (D) creatinine, (E) uric acid, (F) alanine aminotransferase (ALT). Prior to statistical analysis, data were screened for outliers and tested for normality. For normally distributed data, a one-way ANOVA with Tukey's post hoc test was used; for non-normally distributed data, a Kruskal-Wallis test with Dunn's multiple comparisons was used. Mean values are presented as ±SEM. n = 4 mice per group.

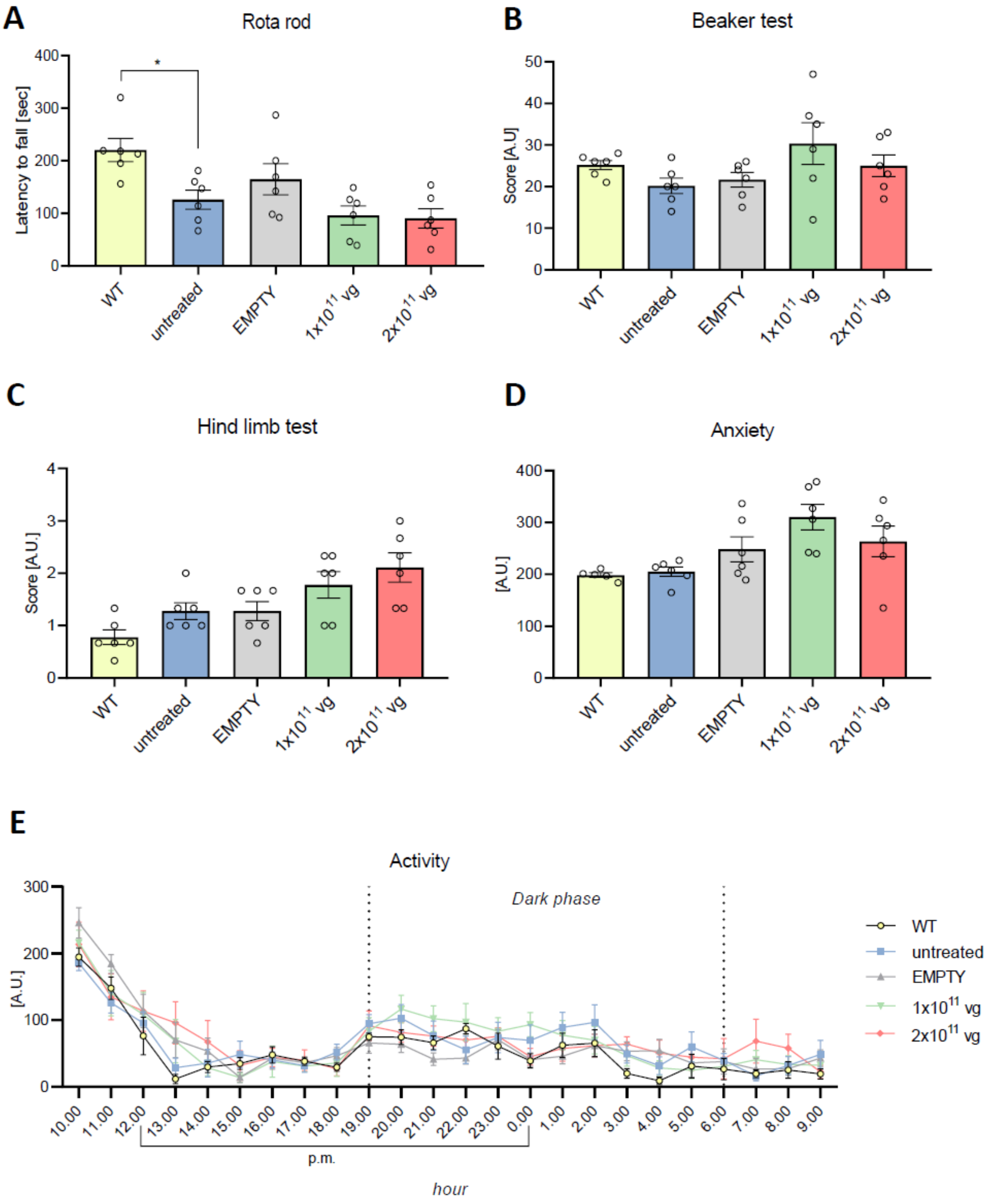

Supplementary Figure 7. Behavioral battery 28 weeks post-injection. Panels show the results from the following tests: (A) Rotarod test, (B) Beaker test, (C) Hind limb reflex test, (D) Activity in the periphery during the Novel environment activity test, and (E) Activity in the center during the Home Cage Activity test. Graphs represent mean values ±SEM. Prior to statistical analysis, data were screened for outliers and tested for normality. For normally distributed data, one-way ANOVA (A, B, D) and two-way ANOVA (E) with Tukey's post hoc test were used. For data that did not meet normality assumptions (C), the Kruskal-Wallis test with Dunn's multiple comparisons was applied. *p-values* are indicated by asterisks (*$p < 0.05$). n = 6 mice per group.

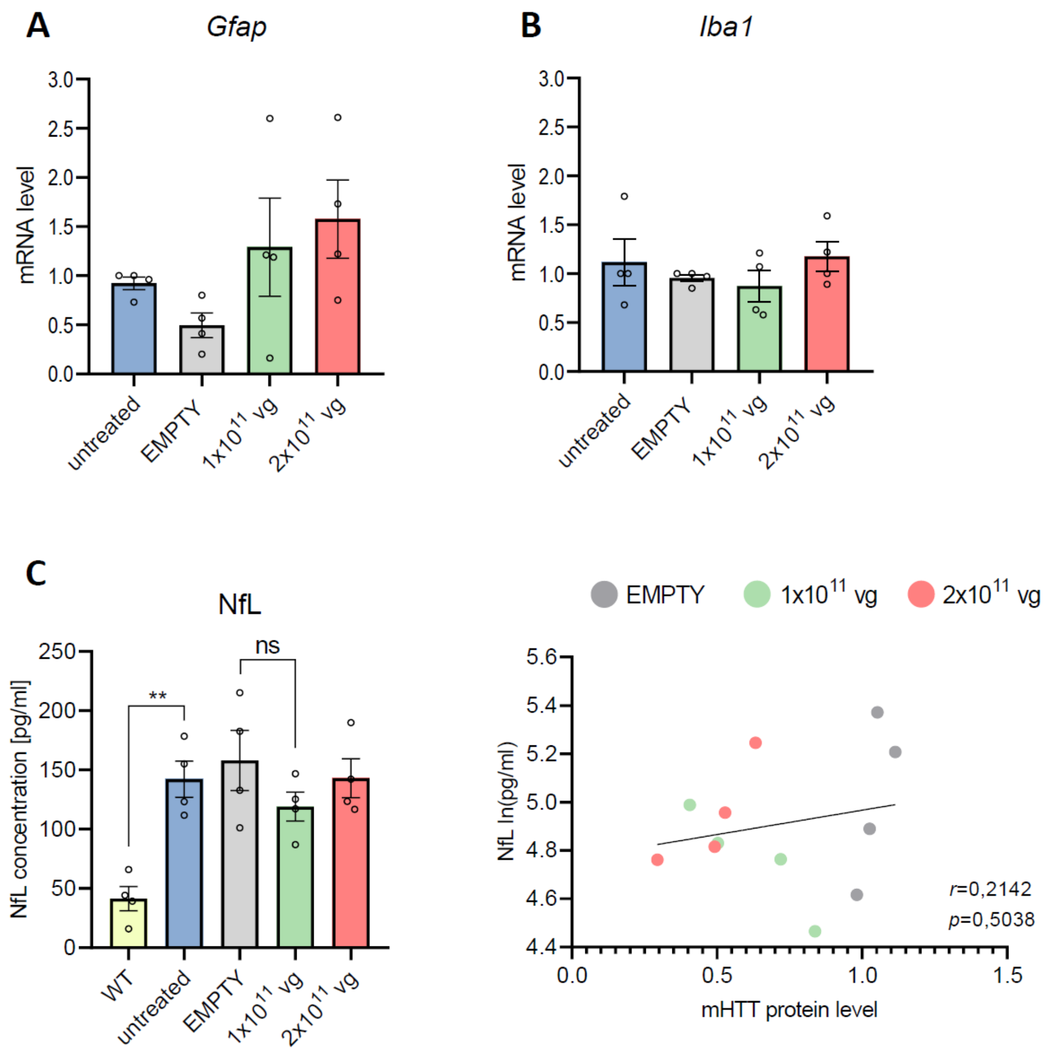

Supplementary Figure 8. Analysis of neuroinflammation markers 28 weeks post-injection of amiR136-13A. (A-B) Analysis of mRNA levels of HD progression markers – Gfap and Iba1. RT-qPCR was performed on RNA isolated from the mouse striata 28 weeks after injection. mRNA levels were normalized to the reference gene *β-actin*. Bars represent mean mRNA levels ± SEM calculated from four biological replicates, each representing the average of the left and right brain hemispheres, with at least three technical replicates per sample. Statistical analysis was performed using one-way ANOVA followed by Tukey's post hoc test, which revealed no significant differences between groups. (C) Serum NfL concentration 28 weeks post-injection and its correlation with mutHTT levels. Serum NfL was measured using the Simoa Assay. In the left panel, bars represent mean NfL concentrations ± SEM calculated from three technical replicates. Statistical analysis was performed using one-way ANOVA followed by Tukey's post hoc test. The *p-value* for the comparison between WT and untreated YAC128 mice is indicated by asterisks (**$p < 0.01$). ns – not statistically significant. The right panel shows the correlation between mutHTT levels (mean value per mouse) in the striatum and serum NfL concentration in the same animals (Pearson's correlation: $r = 0.2142$, $p = 0.5038$). NfL concentrations are presented as natural log-transformed values. n = 4 mice per group.

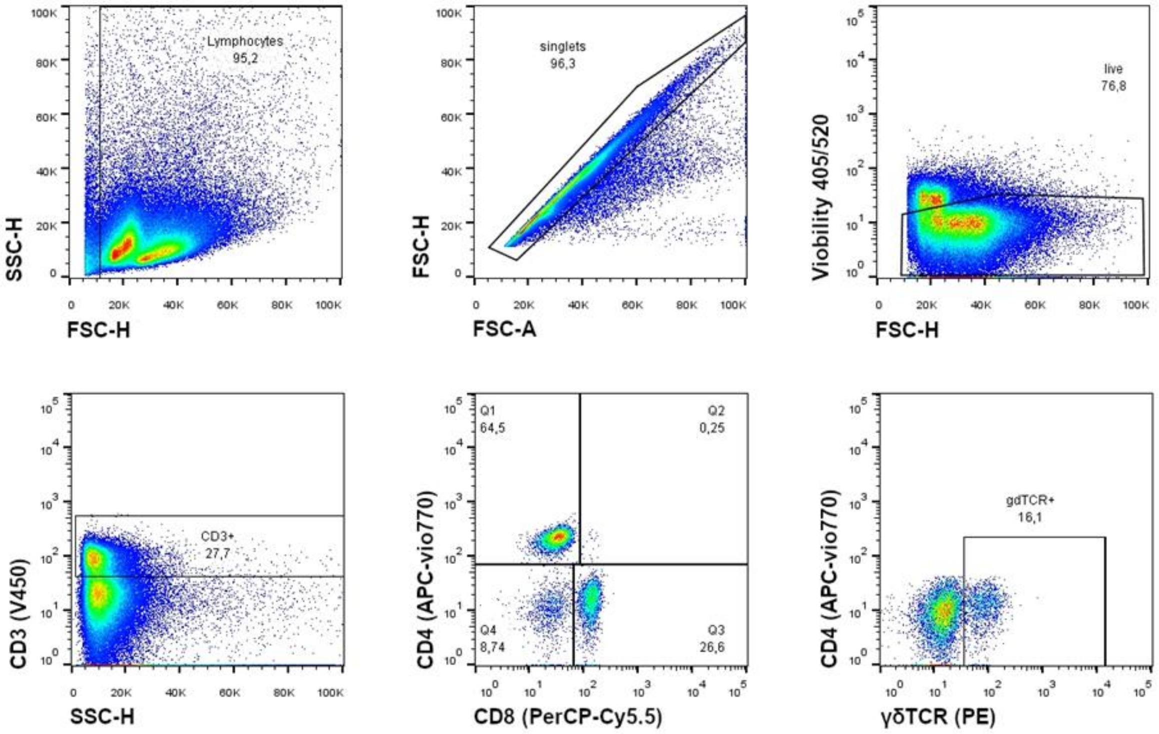

Supplementary Figure 9. Representative gating strategy for T cell subsets in mouse splenocytes. Single cells were first identified, followed by gating on live cells by excluding events positive for the Viability 405/520 dye. Total T cells were defined as CD3$^+$ cells, which were further subdivided into CD4$^+$ and CD8$^+$ subsets. From the CD4$^-$CD8$^-$ population, γδ T cells were identified based on expression of the γδ T cell receptor (γδTCR).

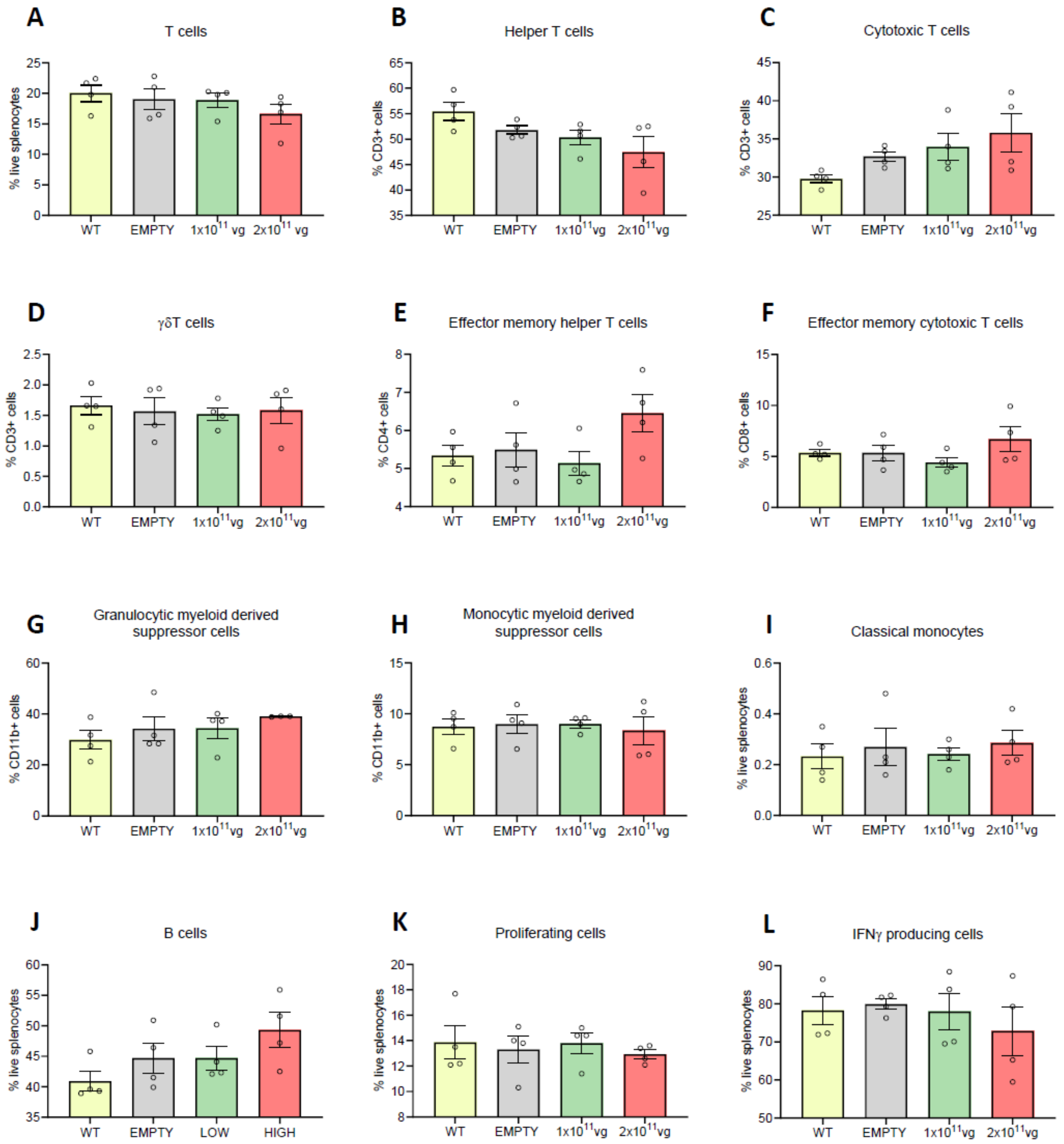

Supplementary Figure 10. Frequencies of immune cell populations in the mouse spleen. (A) T cells ($CD3^+$), (B) helper T cells ($CD3^+CD4^+$), (C) cytotoxic T cells ($CD3^+CD8^+$), (D) γδT cells ($CD3^+CD4^-CD8^-$ $γδTCR^+$), (E) effector memory helper T cells ($CD3^+CD4^+CD44^+CD62L^-$), (F) effector memory cytotoxic T cells ($CD3^+CD4^+CD44^+CD62L^-$), (G) granulocytic myeloid-derived suppressor cells ($CD11b^+GR1^{hi}Ly6C^{lo}$), (H) monocytic myeloid-derived suppressor cells ($CD11b^+GR1^{lo}Ly6C^{hi}$), (I) classical monocytes ($CD11b^+GR1^{hi}SSC^{lo}$), (J) B cells ($CD11c^-CD11b^-B220^+$), (K) proliferating splenocytes ($Ki67^+$), (L) IFNγ-producing splenocytes ($IFNγ^+$). Spleens were harvested 30 weeks post-treatment. Bars represent mean cell percentages ± SEM. Each dot represents an individual mouse (n = 4). Normality of data distribution was assessed using the Shapiro–Wilk test. A p-value < 0.05 was considered indicative of non-normal distribution. For normally distributed data, Brown-Forsythe and Welch's ANOVA with Games-Howell post hoc test were used. For non-normally distributed data, the Kruskal–Wallis test was followed by Dunn's multiple comparisons test.

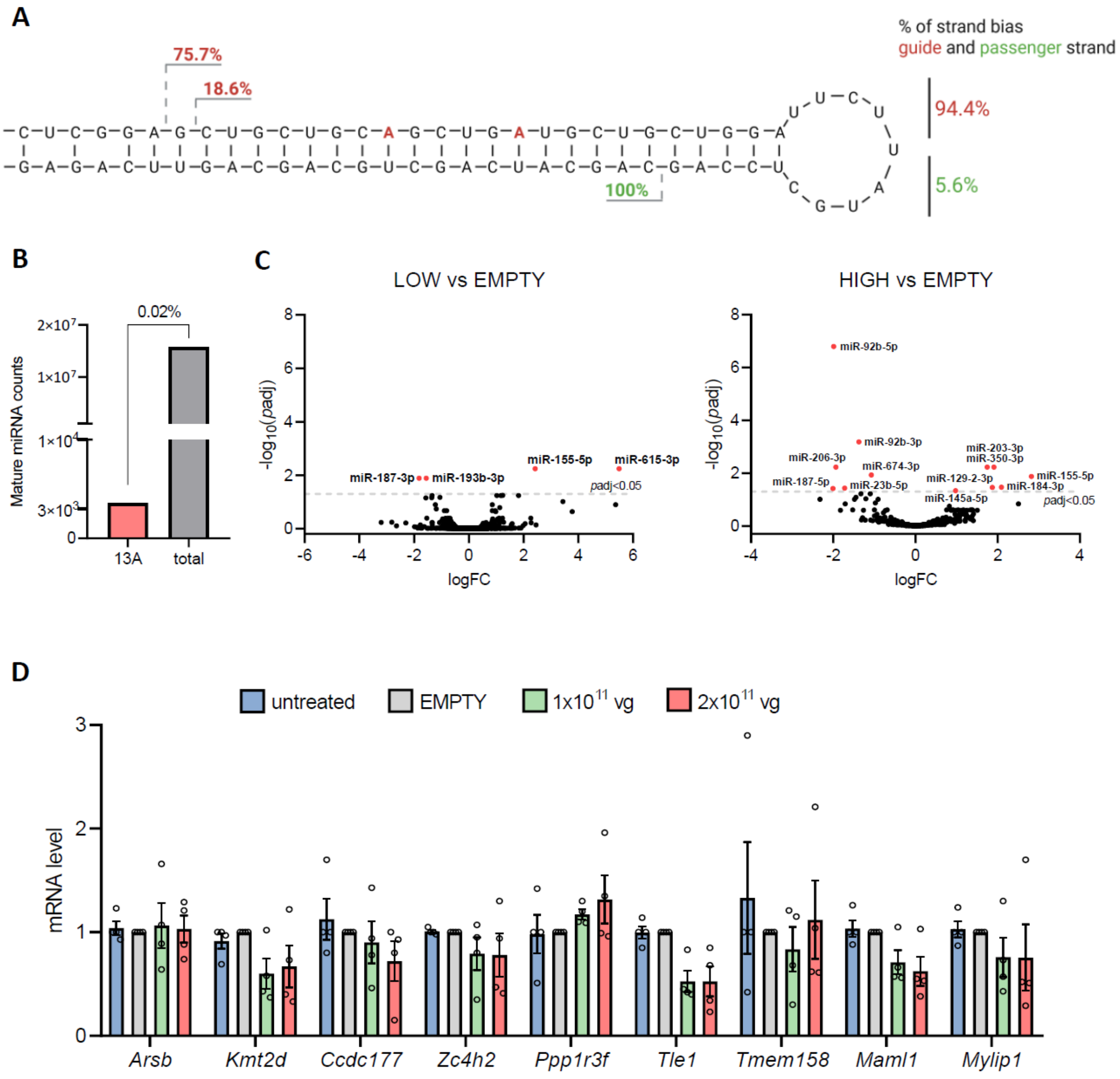

Supplementary Figure 11. Analysis of cellular processing and non-specific effects of amiR136-13A in YAC128 striata 28 weeks post-injection. (A) The most frequently released variants of the 13A guide and passenger strands, grouped according to their 5′ start sites. The guide strand is indicated in red, and the passenger strand is green. Cleavage sites are marked with dashed lines. Percentages indicate the relative abundance of each variant. The overall strand bias is shown on the right. (B) Total read counts for the 50 most highly expressed mature miRNAs following injection. Read counts for all 13A guide strand variants are included. The relative abundance of 13A among the top 50 miRNAs is indicated. (C) Differential gene expression (DEG) analysis comparing mice treated with the amiR136-13A vector (low or high dose) to mice treated with the empty vector. The X-axis shows $\log_2$ fold change (logFC), and the Y-axis shows the $-\log_{10}$ of the adjusted p-value (padj). miRNAs significantly altered (p<0.05) are highlighted in red. (D) Analysis of mRNA levels of predicted off-target genes containing a single mismatch to the 13A guide strand. RT-qPCR was performed on RNA isolated from striata 28 weeks post-injection. mRNA levels were normalized to the reference gene *β-actin*. Bars represent mean ± SEM from four biological replicates and three technical replicates. Values represent the average of both brain hemispheres. Statistical analysis was performed using two-way ANOVA followed by Tukey's post hoc test, which revealed no statistically significant differences between groups (n = 4 mice).

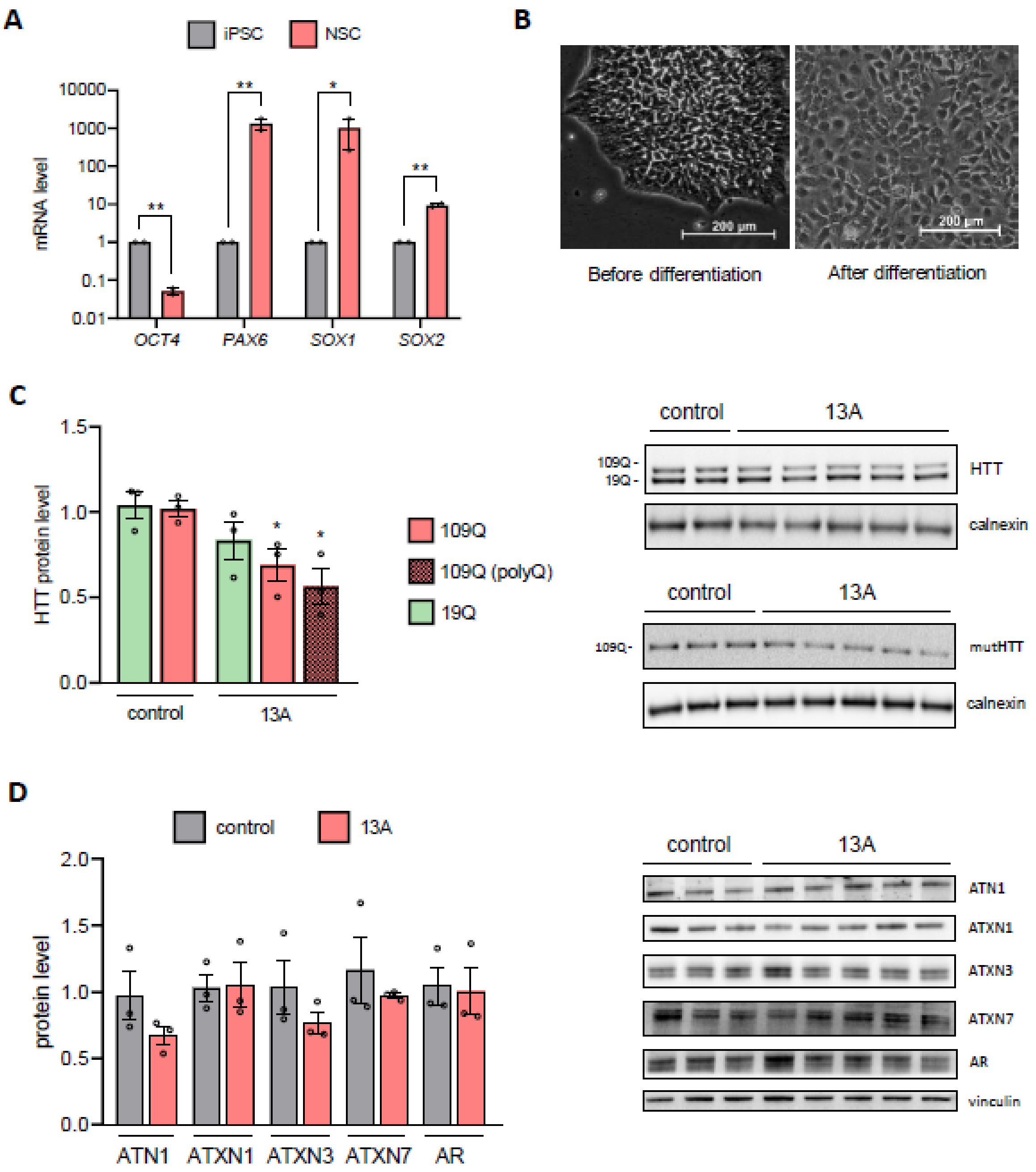

Supplementary Figure 12. Characterization of HD iPSC-derived NSCs and validation of amiR136-13A efficiency prior to transcriptomic analysis. (A) Expression levels of *OCT4, PAX6, SOX1*, and *SOX2* in iPSC-derived NSCs measured by RT-qPCR on day 4 after passaging. Expression was normalized to the levels observed in ND42222 iPSCs. Bars represent mean mRNA levels ± SEMs from two biological and three technical replicates. Statistical analysis was performed using the Student's t-test. (B) Representative images showing the characteristic morphology of iPSCs (before differentiation) and the morphology of NSCs (after differentiation). Scale bar = 200 µm. (C, D) Western blot analysis of (C) HTT, and (D) ATN1, ATXN1, ATXN3, ATXN7, and AR protein levels in HD NSCs differentiated from ND42222 iPSC (109/19Q) at 7 days post-transduction with lentiviral particles containing amiR136-13A (MOI=10). Protein band intensities were normalized to calnexin (C) or vinculin (D). Bars represent mean protein levels ± SEMs from three biological and at least three technical replicates. Biological replicates correspond to three

different clones from the same differentiation. Statistical analysis was performed using the Student's t-test. p-values are indicated by asterisks (*$p < 0.05$, **$p < 0.01$).

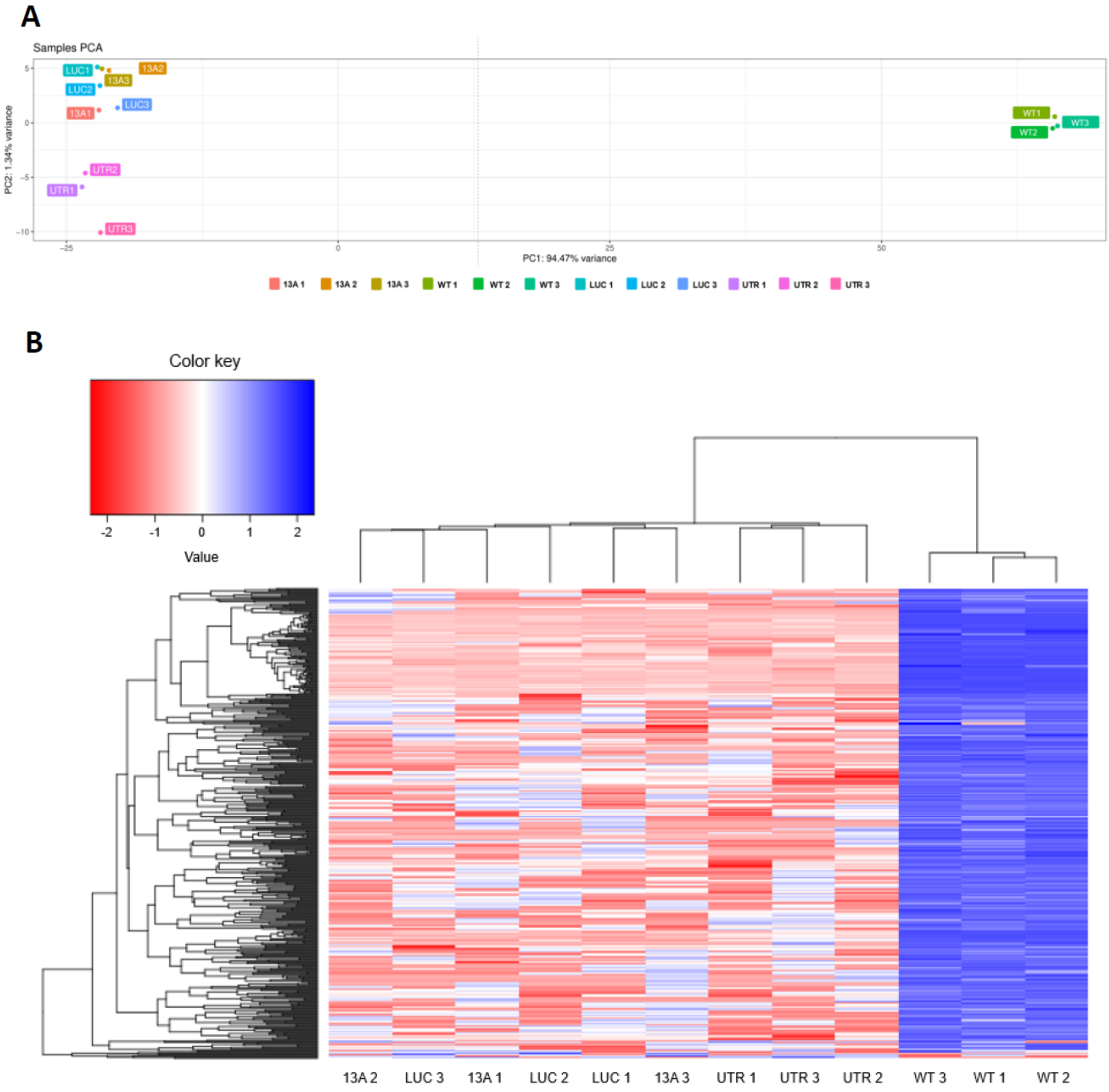

Supplementary Figure 13. Initial RNA-seq data exploration by principal component analysis and heatmap clustering. (A) Principal component analysis (PCA) of HD NSC samples untreated (UTR), amiR136-LUC-treated, amiR136-13A-treated, and isogenic healthy (WT) cells. (B) Heatmap showing hierarchical clustering of samples based on the expression profiles of all detected genes, illustrating the degree of similarity and divergence between experimental groups.

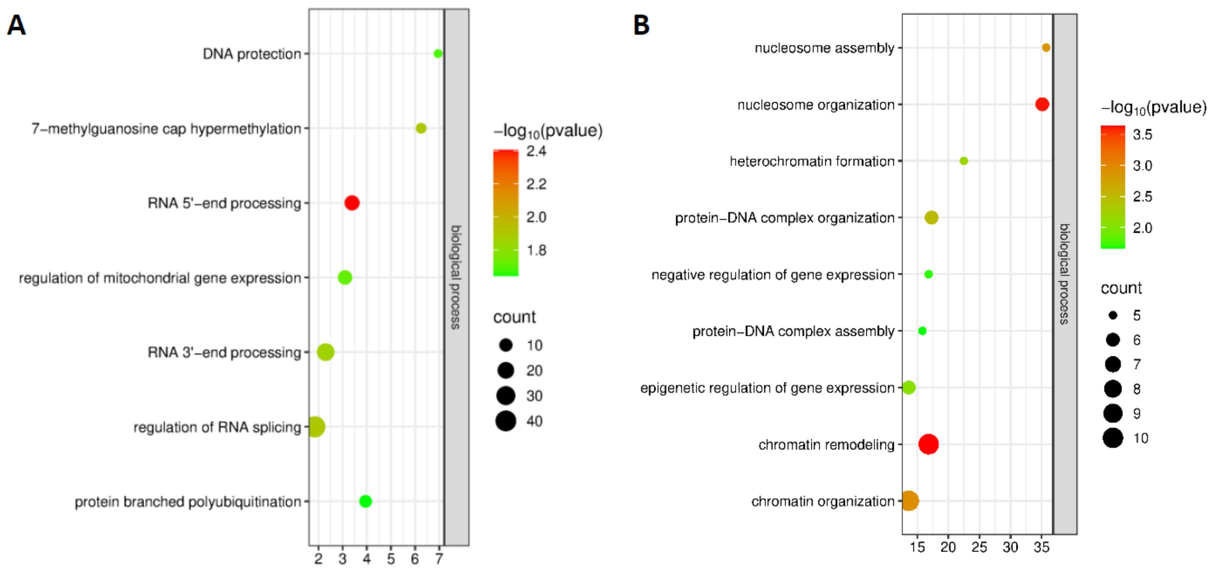

Supplementary figure 14. Bubble plots representing gene ontology enrichment of deregulated genes. (A) Comparison between untreated HD NSCs and healthy isogenic control cells. (B) Comparison between amiR136-13A-treated and amiR136-LUC-treated HD NSCs. n = 3.

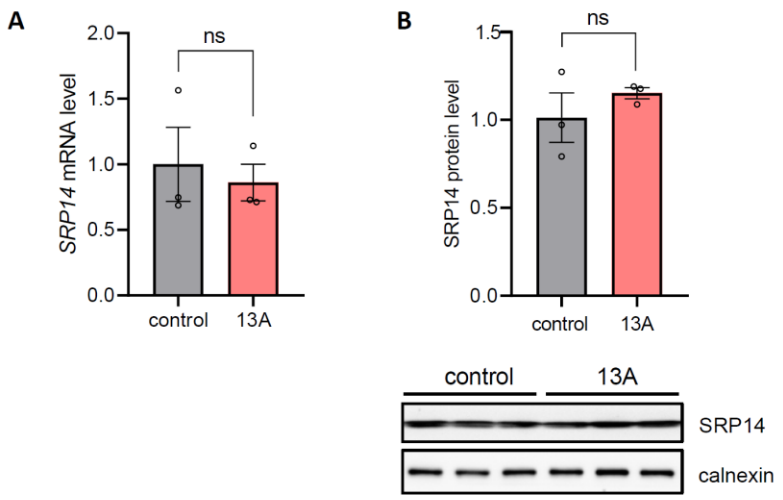

Supplementary Figure 15. Validation of *SRP-14* (off-target) expression in HD NSC after amiR136-13A treatment. (A) RT-qPCR analysis of *SRP-14* mRNA levels. Expression was normalized to the reference gene *EEF2*. Bars represent mean mRNA levels ± SEM from three biological and three technical replicates. Statistical analysis was performed using the Student's t-test. amiR136-LUC was used as a control. (B) Western blot analysis of SRP-14 protein levels in HD NSC at 7 days post-transduction with lentiviral particles containing amiR136-13A or amiR136-LUC control (MOI=10). Bars represent mean protein levels ± SEM from three biological and three technical replicates. Protein band intensities were normalized to calnexin. Statistical analysis was performed using the Student's t-test.

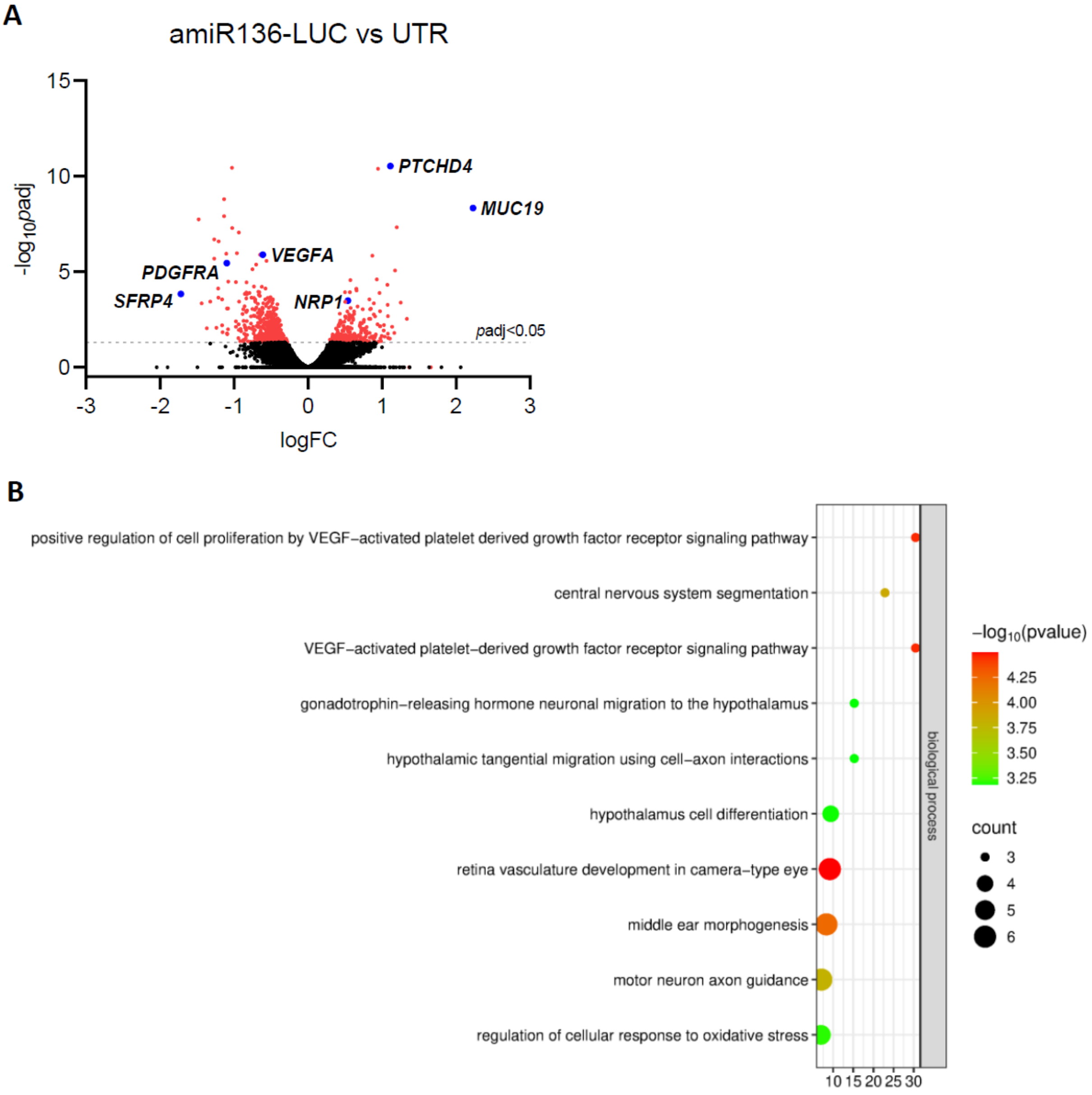

Supplementary Figure 16. Differential gene expression analysis between HD NSCs transduced with amiR136-LUC lentivirus and untreated (UTR) HD NSCs. (A) Volcano plot showing $\log_2$ fold change (logFC) on the X-axis and the negative base-10 logarithm of the adjusted *p*-value ($-\log_{10}$*padj*) on the Y-axis. Genes with statistically significant changes (*padj* < 0.05) are highlighted in red. (B) Bubble plot representing gene ontology enrichment of deregulated genes. n=3.

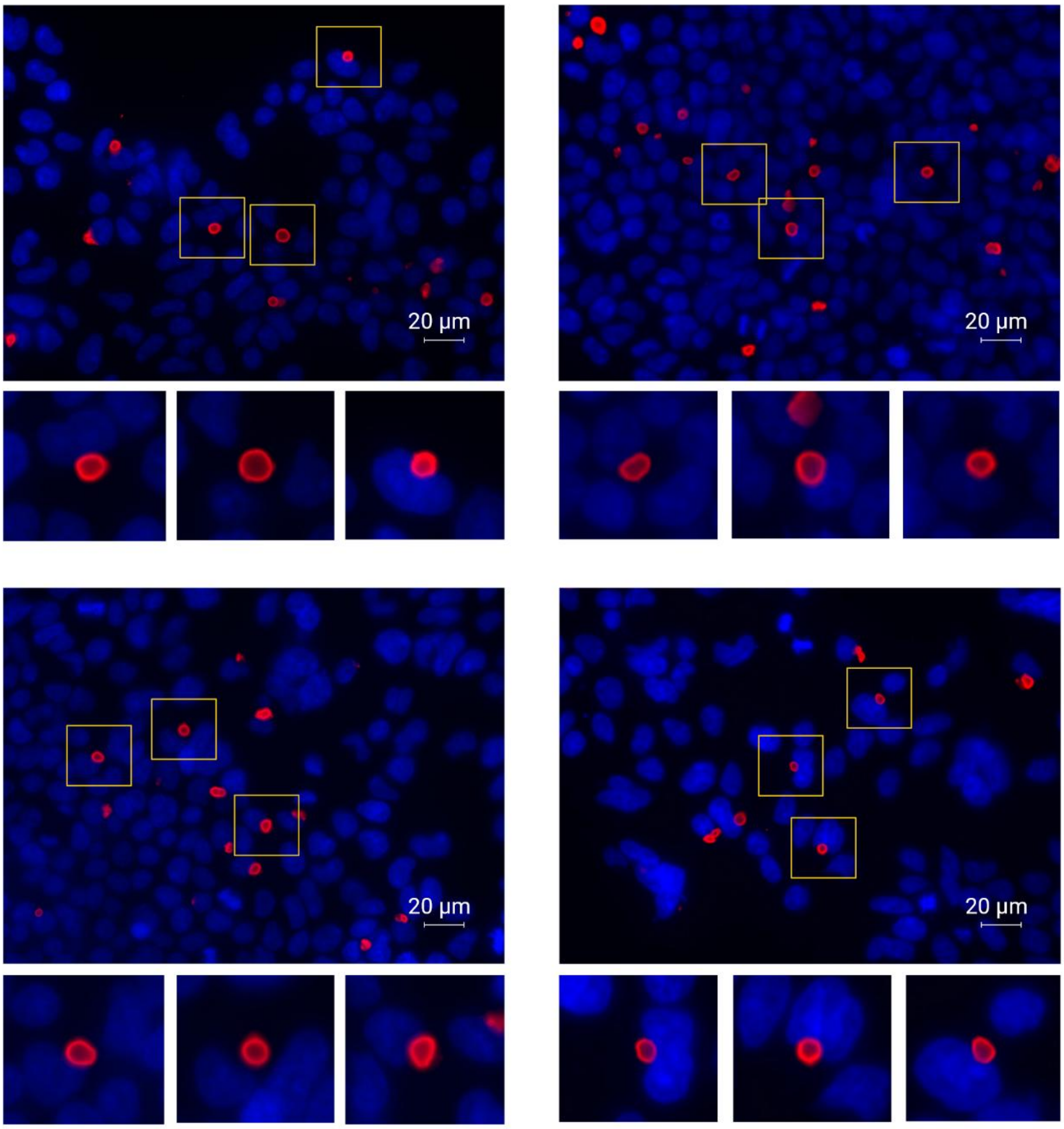

Supplementary Figure 17. Immunohistochemical staining of HEK293 HTT1a cells using anti-FLAG antibody to show HTT1a aggregates (red spots) in control samples. Four representative fields are shown, with selected HTT1a aggregates highlighted in yellow boxes and presented as magnified insets below each panel. Nuclei are counterstained with DAPI (blue). Scale bar = 20 μm.

Supplementary methods

Behavioral tests

Behavioral tests were conducted by experienced personnel blinded to the treatment and genotype of individual animals.

Hind Limb Clasping
Behavioral assessment was performed as previously described (2). Briefly, mice were gripped by the tail to observe hind limb clasping. Scoring was as follows:
0 - hind limbs fully extended and spread apart from the abdomen, toes wide open;
1 - one or both limbs partially brought toward the abdomen but not touching it, toes wide open;
2 - both hind limbs partially advancing and touching the abdomen, but not touching each other;
3 - both hind limbs close to the abdomen and intertwined
Each mouse was evaluated at least twice per day, and the scores were averaged. The Hind Limb Clasping test was performed prior to surgery and at 5, 10, 15, 20, and 28 weeks post-surgery.

Home Cage Activity
Home Cage Activity was used to monitor mouse activity continuously over a 24-hour period and was performed as previously described (3). Mice were housed in conventional cages with bedding, food, and water available *ad libitum*. Activity was recorded for 24 hours, covering a 12-hour light phase and a 12-hour dark phase, using the TSE PhenoMaster system (software version 4.8.9, 2013-4929). Six parameters were measured, including general activity (sum of ambulatory and fine movements), activity in the center of the cage and activity in the periphery, distance traveled per minute, total distance traveled, and sum of vertical movements. Fine movements were defined as repeated beam breaks of the same light barrier, while ambulatory movements were counted as beam breaks detected by alternating beams. Tests were repeated at 10, 20, and 28 weeks after surgery.

Novel Environment Activity
Novel Environment Activity Test was designed to assess general activity levels and exploratory behavior, primarily under stressful conditions. Mice were placed individually in conventional cages without bedding, water, or food. Activity was recorded continuously for 15 minutes to observe acute responses. The same parameters as in the Home Cage Activity test were measured using the same software. Tests were repeated at 10, 20, and 28 weeks post-surgery.

Tissue Collection

Whole spleens were harvested; a small piece was snap-frozen, while the remaining tissue was immediately placed on ice in phosphate-buffered saline (PBS) supplemented with 0.5% bovine

serum albumin (BSA) (Sigma Aldrich) and 2 mM EDTA (Thermo Fisher Scientific). Splenocytes were freshly isolated by mechanical dissociation through a 70 µm cell strainer. Red blood cells were lysed using eBioscience RBC Lysis Buffer (Thermo Fisher Scientific) for 4 minutes at room temperature. The remaining cells were washed with 20 mL PBS containing 0.5% BSA and 2 mM EDTA, then filtered through a 40 µm cell strainer. Cell counts were performed using a TC20 automated cell counter (Bio-Rad).

Staining and Flow Cytometry

One million viable splenocytes were placed on ice and washed twice with PBS. Cells used for cytokine staining were restimulated by resuspension in 200 µL RPMI 1640 medium supplemented with 10% FBS (EurX), 1% penicillin-streptomycin (Thermo Gibco), and 1x Cell Stimulation Cocktail with protein transport inhibitors (Thermo Fisher Scientific). Cells were incubated at 37°C 5% CO_2 for 4 hours. BD GolgiStop (BD) was added at a 1:100 dilution during all subsequent incubations of restimulated samples. Cells were stained with Viability 405/520 dye (Miltenyi Biotec) at a 1:1000 dilution in PBS for 30 minutes on ice. Following viability staining, cells were washed twice with PBS supplemented with 0.5% BSA and 2 mM EDTA. Cells were then blocked with TruStain FcX PLUS (1:100, BioLegend, cat. 156604) and incubated for 30 minutes on ice with antibodies prepared in PBS with 0.5% BSA and 2 mM EDTA for the staining of surface antigens. After staining, cells were washed twice with PBS containing 0.5% BSA and 2 mM EDTA, then fixed and permeabilized in the Foxp3/Transcription Factor Staining Buffer Set (Thermo Fisher Scientific) for 45 minutes. They were subsequently washed twice with 1x permeabilization buffer (Thermo Fisher Scientific). After fixation, cells were incubated with the antibodies prepared in 1x permeabilization buffer for 45 minutes on ice to stain for intracellular proteins. After incubation, cells were washed twice in 1x permeabilization buffer and then resuspended in 250 µL of the same buffer. They were analyzed using a Guava 12HT flow cytometer (Cytek Biosciences). Data were analyzed with FlowJo v10 software. All antibodies used are listed in Supplemental Table 5.

Biochemistry

Blood was collected from mice 20 weeks post-surgery by cardiac puncture following euthanasia with an overdose of isoflurane (Isotek, LABORATORIOS KARIZOO). Sampling was performed using a 1 mL syringe with a 30-gauge needle. Blood samples were left at room temperature on the benchtop for 30 minutes, then centrifuged at 1300 x g for 15 minutes. Fresh serum was collected and used for analysis. Serum concentrations of glucose, cholesterol, creatinine, uric acid, alanine aminotransferase, and aspartate aminotransferase were measured using a Mindray BS-120 automated clinical chemistry analyzer.

References

1. Kotowska-Zimmer,A., Przybyl,L., Pewinska,M., Suszynska-Zajczyk,J., Wronka,D., Figiel,M. and Olejniczak,M. (2022) A CAG repeat-targeting artificial miRNA lowers the mutant huntingtin level in the YAC128 model of Huntington’s disease. *Molecular Therapy - Nucleic Acids*, **28**, 702–715.

2. Zhu,J.-W., Li,Y.-F., Wang,Z.-T., Jia,W.-Q. and Xu,R.-X. (2016) Toll-Like Receptor 4 Deficiency Impairs Motor Coordination. *Front Neurosci*, **10**, 33.

3. Hübener,J., Casadei,N., Teismann,P., Seeliger,M.W., Björkqvist,M., Von Hörsten,S., Riess,O. and Nguyen,H.P. (2012) Automated Behavioral Phenotyping Reveals Presymptomatic Alterations in a SCA3 Genetrap Mouse Model. *Journal of Genetics and Genomics*, **39**, 287–299.
